# eIF5A coordinates the transcription and translation of its target genes

**DOI:** 10.1007/s00018-026-06252-8

**Published:** 2026-05-22

**Authors:** Marina Barba-Aliaga, Lianqi Chi, Samoa Prieto-Díez, Jordi Planells, José García-Martínez, José E. Pérez-Ortín, Paula Alepuz

**Affiliations:** 1 Instituto de Biotecnología y Biomedicina (Biotecmed), Universitat de València, Burjassot, València 46100 Spain; 2 Departamento de Bioquímica y Biología Molecular, Facultad de Biología, Universitat de València, Burjassot, València 46100 Spain; 3 Departamento de Biología Celular, Biología Funcional y Antropología Física, Facultad de Biología, Universitat de València, Burjassot, València 46100 Spain; 4 Departamento de Genética, Facultad de Biología, Universitat de València, Burjassot, València 46100 Spain; 5 Present Address: Department of Molecular and Cellular Biology, Faculty of Arts and Sciences, Harvard University, Cambridge, MA 02138 USA

**Keywords:** eIF5A, Gene expression, Translation elongation factor, Transcription-translation crosstalk, Translation efficiency, Polyprolines

## Abstract

Maintaining balanced cellular protein levels requires precise control of gene expression and effective coordination between the various stages of the process, from transcription to translation. In recent years, several components of the translation apparatus have been found in the nuclei of various eukaryotes, where they regulate transcription, mRNA processing or export, thereby integrating different stages of gene expression. eIF5A is an essential and evolutionarily conserved translation elongation factor that is involved in viral infection and in the development of diseases such as cancer and neurodevelopmental disorders. eIF5A promotes translation elongation by binding to ribosomes that stall at codons encoding problematic amino acids for peptide bond formation, such as consecutive prolines, also known as polyproline motifs. Although eIF5A shuttles between the nucleus and cytoplasm, its specific nuclear roles remain poorly defined. Here, we demonstrate that nuclear yeast eIF5A binds to chromatin and represses gene transcription by preventing the binding of RNA polymerase II. Importantly, chromatin binding and transcriptional repression by eIF5A have a higher impact on genes encoding its own translational targets. The presence of polyproline motifs in genes imposes both translation and transcriptional control by eIF5A. Furthermore, eIF5A's active engagement in cytoplasmic translation is necessary for its role in repressing transcription. Our results suggest that eIF5A coordinates gene expression by promoting the cytoplasmic translation of specific genes while repressing their transcription in the nucleus, thus ensuring efficient final protein synthesis.

**Supplementary Information:**

The online version contains supplementary material available at 10.1007/s00018-026-06252-8.

## Introduction

Eukaryotic translation initiation factor 5A (eIF5A) is an essential, evolutionarily conserved protein with puzzling biological functions. eIF5A is the only known protein post-translationally modified by hypusination, which involves the addition of the polyamine spermidine via the action of two enzymes: deoxyhypusine synthase (DHPS) and deoxyhypusine hydroxylase (DOHH). eIF5A promotes cell proliferation and development, and is involved in apoptosis, autophagy and cytoskeleton organization, as well as maintaining mitochondrial activity. Non-physiological levels of eIF5A or its hypusination enzymes have been linked to diseases such as diabetes, chronic inflammation, altered immune responses, and various cancers. Furthermore, eIF5A promotes viral infection. A decline in hypusinated eIF5A levels has been associated with cellular aging in yeast, flies, mice and humans. In contrast, enhanced hypusination of eIF5A through dietary supplementation with spermidine appears to preserve mitochondrial and cognitive function during aging in flies and mice. Additionally, variants in eIF5A, DHPS and DOHH genes cause rare inherited neurodevelopmental disorders (see [1–8] for reviews).

Some of these diverse eIF5A biological functions rely on its role as a translation elongation factor, which is required for synthesizing specific proteins. Thus, although not necessary for each round of translation elongation, hypusinated-eIF5A binds to ribosomes and promotes the formation of peptide bonds between amino acids known to be poor substrates for this reaction that otherwise stall translation, such as consecutive prolines (polyPro motifs) but also combinations of proline, glycine and charged amino acids [9–11].

Despite being a translation elongation factor, eIF5A is not exclusively confined to the cytoplasm, as it shuttles between this compartment and the nucleus. In mammalian cells, the nuclear export of eIF5A preferably requires exportin 4 (Xpo4) [12, 13], and less frequently the nuclear export receptor Xpo1/CRM1 [14]; whereas in yeast cells, the export relies on Pdr6 [15, 16]. Remarkably, post-translational modifications dictate the subcellular distribution of eIF5A: hypusination and sumoylation promote cytoplasmic localization, while acetylation favors nuclear localization [17–19].

The partial nuclear localization of eIF5A is similar to that of other translation factors, which are among the most abundant proteins in the cell, and is likely to be related to additional functions in mRNA metabolism. In this regard, eIF5A is involved in the nuclear export of unspliced HIV-1 viral mRNA through its interaction with HIV-1 Rev, hence impacting the replication, transcription and translation of the HIV-1 genome in mammalian cells [20–22]. eIF5A also mediates the export of *NOS2* mRNA and *TSC2* mRNA in mammalian models [23, 24]. Furthermore, eIF5A has been found to be associated with RNA polymerase II (RNA Pol II) in the nuclei of precursor neurons [25]. Under hypoxic conditions, the nuclear eIF5A2 isoform binds to the promoter region of the hypoxia-inducible factor 1α (HIF-1α), although its role in HIF-1α activation is not fully clear [26, 27]. The eIF5A2 isoform also indirectly regulates the transcription of ageing genes in human neuroblastoma cells by modulating transcription factors associated with the unfolded protein response [28].

Although transcription and translation are considered independent processes due to their distinct molecular mechanisms, timing and sites of action, several factors have been proposed to coordinate them, however, the crosstalk between the two processes is poorly understood [29, 30]. Here, we describe a novel function of nuclear yeast eIF5A in transcriptional regulation. By binding to specific coding regions, eIF5A attenuates the recruitment of RNA Pol II to tightly control the expression of the eIF5A translation-dependent mRNAs. Furthermore, we demonstrate that this transcriptional regulation is driven by the eIF5A-dependent motifs, which allow for positive translation regulation in the cytoplasm while driving negative transcriptional regulation in the nucleus, resulting in the fine-tuning of translation efficiencies. These findings expand our understanding of the role of eIF5A in coordinating the various stages of gene expression.

## Materials and methods

### Yeast strains, plasmids, growth conditions and materials

*S. cerevisiae* strains and plasmids used are listed in Supplementary Tables S1 and S2 respectively. For all the experiments carried out *S. cerevisiae* cells were grown in liquid YPD (2% glucose, 2% peptone, 1% yeast extract).

For detailed procedures of plasmids and strains generation and all materials used see Supplementary Materials and methods.

### RT-qPCR analysis

For the analysis of the mRNA levels, total RNAs were isolated from yeast cells following the protocol described in [31] The reverse transcription and quantitative PCR reactions were performed as detailed in [32]. Endogenous *ACT1* mRNA levels were used for normalization. At least three biological replicates of each sample were analyzed, and the specific primers designed to amplify gene fragments of interest are listed in Supplementary Table S3. More details can be found in Supplementary Materials and methods.

### Western blotting

For yeast protein content analysis by western blotting we followed the protocol described in [33]. Samples were run on SDS-PAGE gels, transferred, and immunoprobed as described in Supplementary Materials and methods. Antibodies used in this study are listed in the Supplementary Table S4. In order to capture variation across all samples, the signal in each lane was normalized to the mean signal across all lanes in a single blot. Then, the resulting signal of bands was normalized against the corresponding G6PDH resulting signal. At least three biological replicates of each sample were analyzed.

### Fluorescence microscopy and analysis

Yeast cells grown to a logarithmic phase in YPD medium were subjected to standard fluorescence and phase contrast microscopy. Fluorescence images were acquired using an Axio Imager Z1 fluorescence motorized microscope (Carl Zeiss, Germany). Images were recorded with an AxioCam MRm digital camera (Carl Zeiss, Germany). 4′,6-Diamidino-2-phenylindole dihydrochloride (DAPI) was used to visualize nuclei. The same exposure times were used to acquire all images and at least three biological replicates of each sample were analyzed. All the imaging analysis was performed on Image J software. At least 100 single cells were scored from three independent experiments. More detailed procedures can be found in Supplementary Materials and methods.

### Chromatin immunoprecipitation

The chromatin immunoprecipitation (ChIP) experiments were performed as previously described [34] with the modifications described in [31]. qPCR was run as described above using the primers listed in Supplementary Table S3. The qPCR amplification data were normalized with the total input DNA value in the corresponding whole cell extract. Immunoprecipitations of eIF5A were made with anti-eIF5A antibody and Dynabeads anti-rabbit IgG; and of RNA Pol II with anti-Rpb1 antibody and Dynabeads Pan Mouse IgG. At least three biological replicates of each sample were analyzed. More detailed procedures can be found in Supplementary Materials and methods.

### ChIP-seq and sequencing analysis

Wild-type yeast cells exponentially grown in YPD at 25 °C were used for ChIP-seq experiments. Libraries for ChIP-Seq were prepared at IRB Barcelona Functional Genomics Core Facility and submitted for single end 50 nt sequencing on a NextSeq2000 (Illumina). More than 4 Gbp of reads were produced, with a minimum of 17 million of single end reads per sample.

Data analyses was performed at the Statistical and Omics Data Analyses facility of the SCSIE-Universitat de València following the pipeline described in Supplementary Materials and methods.

Biological process Gene Ontology (GO) terms overrepresented in genes with the highest eIF5A binding or the highest eIF5A translation dependence measured with the Protein Pause Index (PPI) were identified using Gorilla tool and the resulting list of GO identifiers with their adjusted p-values was submitted to ReviGO software. ReviGO was run with the default semantic similarity threshold to cluster redundant GO terms and return a representative subset.

More detailed procedures can be found in Supplementary Materials and methods.

### Calculation of protein pause index (PPI) and eIF5A translation dependency measured by Ribosome profiling (eIF5Am/wt RiboReads end/start)

The translation dependency of each yeast gene on eIF5A was estimated by calculating the PPI (6278 genes with data for eIF5A binding by ChIP seq). First, the number of the top 43 eIF5A-dependent tripeptide motifs present in the encoded protein amino acid sequence was determined. These motifs cause ribosome pausing when eIF5A is depleted in yeast cells, as described by [10]. Secondly, the number of each motif was multiplied by its pause strength value, as revealed by 5PSeq analysis [10]. Thirdly, the PPI for each gene was obtained as the sum of motifs x strength.

Data from [11] was also utilized to estimate the translation dependency of each gene on eIF5A (5082 genes with data both for ribosome profiling and eIF5A binding by ChIPseq). Firstly, the ratio of ribosome footprinting reads 200 bp upstream and downstream of the stop and start codons, respectively (RiboReads end/start), was calculated for wild-type cells and for cells depleted of eIF5A. Subsequently, the log_2_ of this ratio was determined in eIF5A-depleted cells relative to the ratio in wild-type cells (eIF5Am/wt RiboReads end/start). The interval of data ranged from 3.598 to -8.281. The more negative the value, the greater failure to complete translation by the ribosomes, and consequently, a higher degree of eIF5A translation dependency.

### Determination of individual transcription rates and mRNA levels

To determine the transcription rate in the corresponding strains, a genomic run-on (GRO) was performed as originally described in [35] and modifications made in [36] Briefly, to perform the run-on, one aliquot of each cell culture under the required conditions was resuspended in the appropriate buffer with [α-^33^P]-UTP and incubated at 30 °C for 5 min to allow transcription elongation. Then, radioactive incorporation into nascent mRNA for each yeast gene was measured using home-made macroarray nylon filters [35].

A second aliquot of the same cell culture was used to isolate total RNA and reverse transcribed in the presence of [α-^33^P]-dCTP and, then, labelled cDNA was used to hybridize macroarray filters. Both GRO and cDNA labelled samples belonging to the same sampling were successively hybridized against the same filter. The scanned microarray images from both GRO and cDNA experiments were quantified using Array Vision software (Imaging Research). Subsequent analysis of the data was performed as described [35]. The experiments were always done in triplicate. To calculate synthesis rate (SR) the transcription rates were normalized by the cellular volume (Supplementary Figure S2C) [37].

More detailed procedures can be found in Supplementary Materials and methods.

### Analysis of mRNA stability and decay

For mRNA decay experiments, yeast cultures were grown in YPD at 25 °C until mid-exponential phase and then incubated for 4 h at either 25 °C or 37 °C. To monitor mRNA stability, transcription was inhibited by the addition of thiolutin, from a 1000 × stock in DMSO, to a final concentration of 5 µg/mL. Samples were harvested at the indicated time points (0, 5, 13, 25, 45, 60, 90, and 120 min) after transcriptional shut-off. Cells were collected by rapid centrifugation at 7,000 rpm for 2 min and flash-frozen in liquid nitrogen. Total RNA extraction and RT-qPCR were performed as described in the corresponding sections with a minor modification: DNAse I-treated RNA was reverse transcribed using the PrimeScript RT Reagent Kit (Perfect Real Time) (Takara) to generate cDNA for real-time PCR with random 6-mers and oligo dT primer, following the manufacturer’s instructions. mRNA decay rates and half-lives were calculated by fitting the Log_2_ of the remaining mRNA levels (normalized to *RDN5*) to a linear regression model. Three biological replicates of each sample were analyzed, and the specific primers designed to amplify gene fragments of interest are listed in Table S3.

### Chromatin association assay

The chromatin association assay experiment was performed as previously described in [38], with modifications. Yeast cell cultures were grown in 300 mL of YPD medium at 25 °C to mid-log phase (OD_600_ 0.5). Cells were collected and pellets were processed to break cells as described in Supplementary Materials and Methods. The lysate was divided into two samples: one half was treated with RNase A and RNase T1; and the other half was incubated without RNase. After 1-h incubation at 25ºC, chromatin was isolated by three-times centrifugation at 13,000 rpm for 20 min. Chromatin was solubilized and analyzed by SDS-PAGE and Western blotting against eIF5A, cytoplasmic Pgk1 and nuclear H4 proteins with specific antibodies. At least three biological replicates of each sample were analyzed. More detailed procedures can be found in Supplementary Materials and methods.

### Statistical analysis

All experiments were performed in at least three independent biological replicates. For parametric data, two-group comparisons were analyzed using two-tailed paired Student’s t-tests when comparing different conditions of same cultures/strains, or unpaired Student’s t-tests when comparing independently-grown pairs. For multiple comparisons, a One-way ANOVA followed by Tukey’s post-hoc test was used to compare the behavior of different genes within the same biological samples. Two-way ANOVA followed by Estimated Marginal Means (emmeans) post-hoc tests was used to assess pairwise comparisons between different cultures/strains. For non-parametric microscopy and genomic data, the Kruskal–Wallis test was applied for global comparisons across multiple groups, followed by the Wilcoxon rank-sum test for pairwise comparisons and to establish differences in medians. Statistical significance is shown as the p-value threshold: * *p* < 0.05, ** *p* < 0.01, *** *p* < 0.001.

## Results

### Nuclear eIF5A binds to the chromatin of genes that depend on eIF5A for translation

**Fig. 1:**
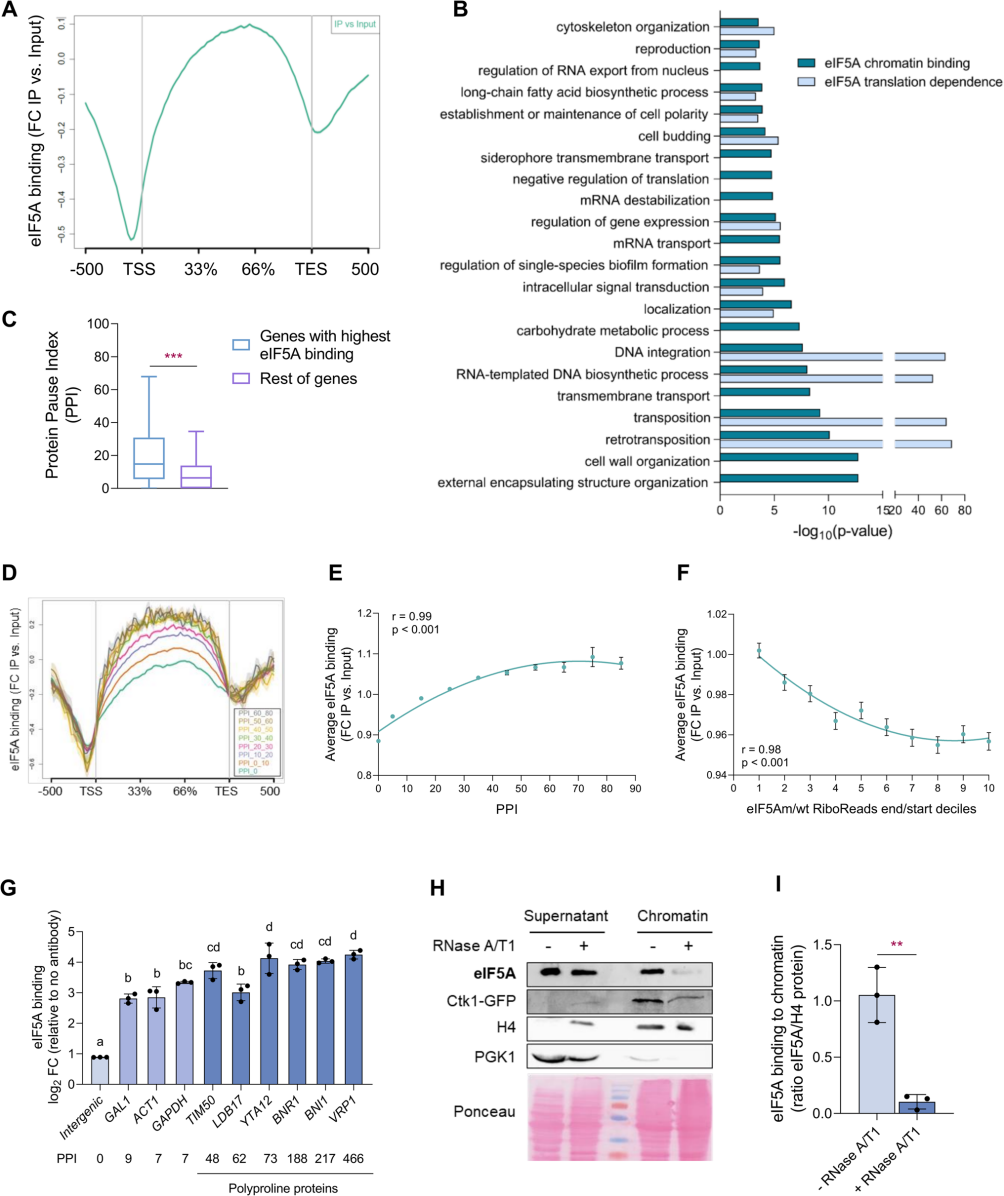
eIF5A binds preferentially to the chromatin of genes whose translation is dependent on eIF5A. **A** Metaplot illustrating the general binding profile of eIF5A to yeast genes obtained by ChIP-seq. Wild-type yeast cells were exponentially-grown in YPD at 25ºC and then ChIP-seq of eIF5A was performed using an anti-eIF5A antibody. The graph shows the global eIF5A distribution in the gene body region, from the transcription start site (TTS) to the transcription end site (TES), corrected by the whole cell extract (metaplot of 5143 genes corresponding to two independent ChIP-seq experiments). **B** Biological process Gene Ontology (GO) terms overrepresented in genes with the highest eIF5A binding or the highest eIF5A translation dependence measured with the Protein Pause Index (PPI). Gene set enrichment analysis was done using the ReviGO software. **C** Distribution of the PPI data from the top genes with the highest eIF5A binding or the rest of genes is presented in box-plots, showing the minimum, first quartile, median, third quartile and maximum. The statistical significance for the pairwise comparisons was estimated by the non-parametric Kruskal–Wallis test followed by the Wilcoxon rank-sum test. **D** Metaplot illustrating eIF5A binding distribution in the gene body region corrected by the whole cell extract for genes included in each group with different PPI intervals (see Supplementary Figure S1A). **E** The average eIF5A binding value relative to input from ChIP-seq analyses is shown for the genes included in each PPI interval group. Experimental data were adjusted to potential trend. The Standard error (SE) and Pearson’s correlation coefficient and the associated significance for the plot is shown. **F** The average eIF5A binding value relative to input from ChIP-seq analyses is shown for genes grouped in deciles according to ribosome profiling data from Schuller et al. (2017). Genes were ranked based on eIF5Am/wt RiboReads end/start and divided into ten deciles containing 508 or 509 genes, and the mean of eIF5A binding value was calculated for each decile (see M&M for full definition of eIF5Am/wt RiboReads end/start). Experimental data were adjusted to a potential trend. Standard error (SE), Pearson’s correlation coefficient, and the associated significance are shown. **G** ChIP analysis of eIF5A recruitment in wild-type cells exponentially-grown in YPD at 25ºC. ChIP of eIF5A was performed using an anti-eIF5A antibody. The immunoprecipitated DNA was used to quantify the binding to the different genes by qPCR using primers designed for amplification in the specific ORF regions. The percentage of the signal obtained in each ChIP sample with respect to the signal obtained with the DNA from the corresponding whole cell extract was calculated and represented as log_2_ relative to signal of non-antibody sample. Statistical significance was determined using a one-way Anova test followed by a Tukey post-hoc test, where different letters mean that values are statistically different. **H** Chromatin association of eIF5A depends on RNA. Wild-type yeast cells containing a CTK1-GFP genomic fusion and exponentially grown in YPD at 25ºC were lysed and then treated or not with an RNase A/T1 mix. After this, chromatin was purified as described in M&M and proteins in the cytoplasmic and chromatin fractions were analyzed by Western blotting using antibodies against eIF5A, GFP, nuclear histone H4 and cytoplasmic Pgk1 proteins. Ponceau staining is shown as protein loading control. **I** Quantification of eIF5A protein levels, with respect histone H4 protein levels for experiments in (**H**). It is shown the mean of eIF5A/H4 ratio of chromatin-associated proteins ± SD from three independent experiments. Statistical significance was determined using a two-tailed paired Student’s t-test of non-treated relative to RNase treatment samples. For all experiments *p < 0.05, **p < 0.01, ***p < 0.001 and n.s means no significant differences

Although eIF5A shuttles between the nucleus and cytoplasm in mammalian and yeast cells, its nuclear functions and putative DNA binding properties still remain unclear. Therefore, we aimed to address whether eIF5A binds to DNA chromatin of specific genes. To answer this question, we performed ChIP-seq experiments in wild-type *Saccharomyces cerevisiae* cells exponentially grown in glucose-based media using a specific eIF5A antibody. Our results revealed that the cross-linking procedure trapped eIF5A on the chromatin and metagene analysis of two biological replicates demonstrated that this binding occurs in transcribed regions (Fig. 1A). We examined the functional categories (GOs) enriched in genes showing the strongest eIF5A binding. We found enrichment in categories including “cell wall organization”, “bud growth”, “actin filament organization”, “cytoskeleton organization”, and “filamentous growth” among others (Fig. 1B). Interestingly, when comparing these GOs of genes with high eIF5A binding to those of genes whose translation relies on eIF5A, as they contain a significant number of eIF5A-dependent motifs such as those with three or more consecutive prolines [10, 11, 39], we found that an important number (13 out of the 22 GOs) were coincident.

To shed light into this, and given that the number of experimentally proven eIF5A direct translational targets is relatively low, we classified the yeast genes according to their putative eIF5A-dependence for translation. First, we defined a Protein Pause Index (PPI) for each gene by using the strength pause values obtained for the top 43 eIF5A-dependent tripeptides revealed by 5PSeq analysis [10]. The three top tripeptides among these were KPP, PPP and PGW, with pause scores of 8.982, 7.032 and 6.410 respectively (signal of stalled ribosomes under eIF5A depleted conditions vs. non-depleted); while the rest were mostly combinations of proline, glycine and/or acid or basic amino acids [10]. The PPI was calculated for each gene as the sum of each of these top 43 eIF5A-dependent tripeptides contained in its corresponding amino acid sequence, multiplied by its specific strength pause value. The higher the PPI for the gene, the more likely that translation of the corresponding mRNA will stall upon eIF5A deficiency. The yeast proteome (data from 6714 proteins) showed an average of 2.9 motifs/protein and an average PPI value of 10.84. A GO search according to the PPI showed that several of the categories with the strongest DNA binding of eIF5A are also functional categories over-represented in those genes with the highest eIF5A requirement for translation (Fig. 1B). Moreover, we found a positive and statistically significant difference in PPI values between genes with highest eIF5A binding and the rest of the genome (Fig. 1C).

We further analyzed the relationship between eIF5A chromatin binding and their eIF5A-dependence for translation by classifying the entire yeast genome into 10 groups showing different PPI intervals (Supplementary Figure S1A). Notably, genes with the highest PPI values exhibited higher levels of eIF5A association to their gene body compared to those with the lowest PPI values (Fig. 1D). In this line, we observed a progressive increase in eIF5A binding as the PPI value increased until reaching a saturation at PPI values above 60, proportional to the presumed eIF5A-dependence for translation (Fig. 1E and Supplementary Figure S1B). To discard the effect of possible outliers when using gene groups sorted by PPI intervals, and therefore with different number of genes in the groups, eIF5A binding was also determined for PPI groups sorted into bins based on PPI value, but with each containing an equal number of genes. Again, a strong positive correlation was observed between eIF5A binding and the PPI value (Supplementary Figure S1C).

Next, to validate the use of the PPI index as a measure of translation dependency on eIF5A, we compared it with the eIF5A translation dependency revealed by ribosome profiling data obtained in wild-type cells and in cells depleted of eIF5A, as shown in [11]. First, we calculated the ratio of ribosome footprinting reads 200 bp upstream and downstream of the stop and start codons respectively (RiboReads end/start). Then, we took the log₂ of this ratio in eIF5A-depleted cells relative to the ratio in wild-type cells (eIF5Am/wt RiboReads end/start). The lower the eIF5Am/wt RiboReads end/start value, the greater the eIF5A dependency on translation; implying that fewer translating ribosomes reach the end of the coding sequences under eIF5A depletion. Accordingly, bona fide eIF5A translation targets such as *TIM50*, encoding an essential component of the TIM23 complex translocase in mitochondria [40], *VRP1*, encoding a proline-rich protein involved in cytoskeletal organization and cytokinesis [9], or *BNI1,* a formin required for the formation of polarized actin cables and member of the cellular polarisome [39], showed some of the lowest eIF5Am/wt RiboReads 200end/200start values (-7.6, -6.4 and -5.5, respectively). We then compared the eIF5A translation dependency, as measured by ribosome profiling, with PPI values, observing a good correlation between the two parameters for the 5,082 genes for which ribosome profiling data was available (Supplementary Fig. s1D). Moreover, in line with our previous analysis, eIF5A binding (ChIP-seq) was higher for genes with greater estimated eIF5A translation dependency by ribosome profiling (Fig. 1F).

Consistently with the above ChIP-seq results, we further confirmed the presence of eIF5A at the locus of genes with high PPI by ChIP-qPCR experiments (Fig. 1G and Supplementary Figure s1E). We found a significant binding of eIF5A to all the tested gene ORFs, compared to an intergenic region, where the signal was almost absent as expected from the general profile (Fig. 1D). However, the genes with high PPI values, including several confirmed translational targets of eIF5A such as *TIM50*, *VRP1*, *BNR1* (formin homologous to Bni1) or *BNI1*, showed significantly higher levels of eIF5A association compared to those with low PPI values (approximately twofold signal).

The association of eIF5A with specific gene bodies could be mediated by DNA or RNA binding. The fact that eIF5A ChIP-seq signals are higher at the 3' ends of transcribed regions (see Fig. 1A and D) suggests that eIF5A binding could occur through interaction with the emerging mRNA. To further investigate this, we performed chromatin purification experiments on yeast cells that had been previously lysed and then incubated with a buffer with or without RNases. Proteins associated with chromatin were then detected by Western blotting. Treatment with RNase led to a significant decrease in the levels of chromatin-associated eIF5A (Fig. 1H), as well as those of the RNA Pol II serine 2 kinase Ctk1, whose chromatin association is known to be strongly influenced by nascent RNA [38]. Conversely, RNase treatment did not interfere with histone H4 binding to DNA, as expected (Fig. 1H). The eIF5A/H4 ratio of chromatin-associated proteins was more than fourfold higher (*p*-value < 0.05) in samples of chromatin purified without RNase pretreatment (Fig. 1I).

Altogether, these results confirm that nuclear eIF5A is bound to the ORF of many genes and that eIF5A binds preferentially to the coding regions of genes encoding target mRNAs requiring this factor for their translation. Moreover, eIF5A association to chromatin is highly dependent on RNA.

### Deficiency of eIF5A increases the transcription and mRNA levels of the genes it binds to, and which require it for their translation

The interaction of eIF5A with chromatin suggests that this protein plays a role in transcriptional regulation and mRNA synthesis. To elucidate the potential effects of eIF5A depletion on mRNA synthesis and metabolism, we determined the synthesis rate (SR) and mRNA amount (RA) of the entire yeast genome using the Genomic Run-On (GRO) method [35]. We used a wild-type strain and the temperature-sensitive strain *tif51A-1* (carrying a single Pro83 to Ser mutation), since eIF5A is an essential protein [41, 42]. The two isogenic yeast strains were grown in glucose-based medium to early exponential phase at permissive temperature (25 °C) and then transferred to restrictive temperature (37 °C) for 4 h. At this point, eIF5A protein levels were almost undetectable in *tif51A-1* cells at 37 °C (Supplementary Fig S2A), and the growth rate was decreased (Supplementary Fig S2B,C), without affecting mutant cells viability [39].

**Fig. 2:**
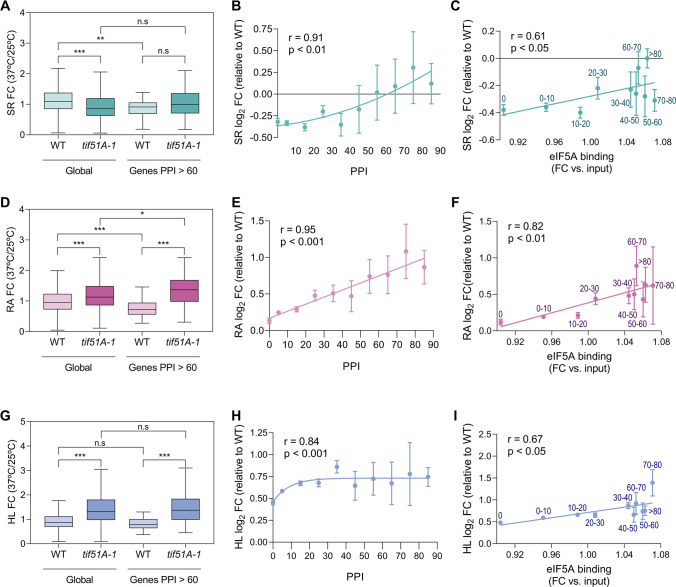
Changes in synthesis rate (SR) and RNA amount (RA) upon eIF5A depletion correlate with the degree of eIF5A-dependency for translation and with the binding of eIF5A to chromatin. Wild-type and *tif51A-1* mutant yeast strains were grown in YPD medium at 25ºC until early exponential phase and then transferred to 25ºC and 37ºC for 4 h. Then cells were collected and processed as described in M&M for GRO assay. **A**,**D**,**G** Genomic data of SR (**A**), RA (**D**) and mRNA half-lives (HL) (**G**) from the global data (4505 genes) and from genes with PPI > 60 (48 genes). Data are presented as the fold change (FC) 37ºC vs. 25ºC from three independent experiments. Graphics show box-and-whisker plots in the style of Tukey, showing the median (middle line), the interquartile range (box), and the inner fences (1.5 × IQR) as whiskers. After applying the non-parametric Kruskal–Wallis test, the statistical significance for the pairwise comparisons was estimated using the Wilcoxon rank-sum test. (B,E,H) Graphs represent the average SR (**B**), RA (**E**), and HL (**H**) for all genes included in each Protein Pause Index (PPI) interval group. Values are given as the log2 fold change (FC) at 37ºC vs. 25ºC ± S.E of *tif51A-1* strain, relative to the corresponding value in the wild-type strain. Experimental data were adjusted to logarithmic (**B**), linear (**E**) or potential (**H**) trends. **C**,**F**,**I** Graphs represent the average SR (**C**), RA (**F**), and HL (**I**) for all genes included in different PPI group (the labels indicate the PPI interval) versus the average eIF5A binding from ChIP-seq analyses for each group of genes. Values represent the log2 fold change (FC) at 37ºC vs. 25ºC ± S.E of *tif51A-1* strain, relative to the corresponding value in the wild-type strain. Experimental data were adjusted to linear trends. **B**,**C**,**E**,**F**,**H**,**I** Pearson’s correlation coefficient and the associated significance for the plots are shown

We first analyzed all the yeast protein-coding genes (4,505 genes) and found that eIF5A depletion reduced global transcription (Fig. 2A left), as expected for a reduction in growth rate [43], but increased total RNA (Fig. 2D left). mRNA half-life estimations revealed a significant stabilization of most transcripts in *tif51A-1* cells (Fig. 2G), indicating that global mRNA stabilization is the main contribution to the increase in RA under eIF5A deficiency.

To study the changes occurring in the set of eIF5A translation-dependent genes, we established a target group of genes in which their PPI was above 60 (48 genes). These genes were strong candidates to be dependent on eIF5A for their translation and included most of the yeast eIF5A-identified targets (Supplementary Fig S1A). We observed that the synthesis rate (SR) of these genes with high eIF5A-dependence followed a markedly different trend compared to global genes upon eIF5A depletion: while the SR of global genes decreased, that of eIF5A-dependent genes tends to increase, although without statistical significance (Fig. 2A). The analysis of the RA among this group of genes showed a more marked and significant RA increase than the global data (Fig. 2D), whereas the analysis of the mRNA half-lives (HL) showed minor differences with regard to the global data (Fig. 2G). Therefore, we identified specific trends for genes predicted to behave as eIF5A translational-dependent, which diverged substantially from the general trend in the *tif51A-1* mutant.

To further investigate this, we used the previous classification of genes according to their PPI values (Supplementary Fig. S1A). We calculated the relative fold change 37 °C vs. 25 °C for each gene in the *tif51A-1* mutant respect to this fold change in the wild-type, and then the mean values of SR, RA and HL and plotted them against the PPI values and the corresponding eIF5A binding values from ChIP-seq for each PPI gene group. These analyses revealed that the relative changes of SR in the eIF5A mutant correlated significantly and positively with the PPI and with the eIF5A binding to chromatin (Fig. 2B,C and Supplementary Figure S2D-G). Consistent with this increase, we observed a proportional increase in RA that was strongly and positively correlated with both the PPI and the eIF5A binding (Fig. 2E,F and Supplementary S2H-K). In contrast, the HL data showed a lower correlation with the PPI and with the eIF5A binding to chromatin (Fig. 2H,I and Supplementary Figure S2L-O). We confirmed that the HL of several mRNAs, which were not predicted to be eIF5A translational targets (low PPI index), increased upon eIF5A depletion (Supplementary Figure S3). These data supported the idea of global mRNA stabilization upon eIF5A depletion, as estimated with genomic data (Fig. 2G).

As eIF5A association with chromatin depends largely on RNA (Fig. 1H,I), we analyzed whether eIF5A binding correlates with gene transcription levels in wild-type or with changes in gene transcription that may occur when cells are shifted from 25 °C to 37 °C to deplete eIF5A protein. We found a modest positive correlation between eIF5A binding to gene bodies and transcription levels (Supplementary Figure S2P) and a very slight correlation between eIF5A binding and SR change during the temperature shift (Supplementary Figure S2Q). Together with our previous results (Supplementary Figure S1C), these observations suggest that the recruitment of eIF5A to chromatin is largely influenced by PPI, and slightly dependent on the synthesis rate.

In sum, our genomic studies concluded that eIF5A depletion reduces the global mRNA synthesis rate, while increasing the overall mRNA levels in yeast cells due to global mRNA stabilization. This increase is possibly due to a compensatory effect, as has been described in both yeast and mammals [43, 44]. Conversely, the levels of mRNAs with both stronger eIF5A-dependence for their translation and eIF5A binding to their chromatin have even a higher relative increase mostly due to the lack of SR decrease. Therefore, these findings suggest a specific role of nuclear eIF5A in the transcriptional repression of genes encoding its target proteins.

### Nuclear localization via an N-terminal NLS and cytoplasmic hypusination of eIF5A are critical for transcriptional repression of eIF5A translation-dependent genes

**Fig. 3:**
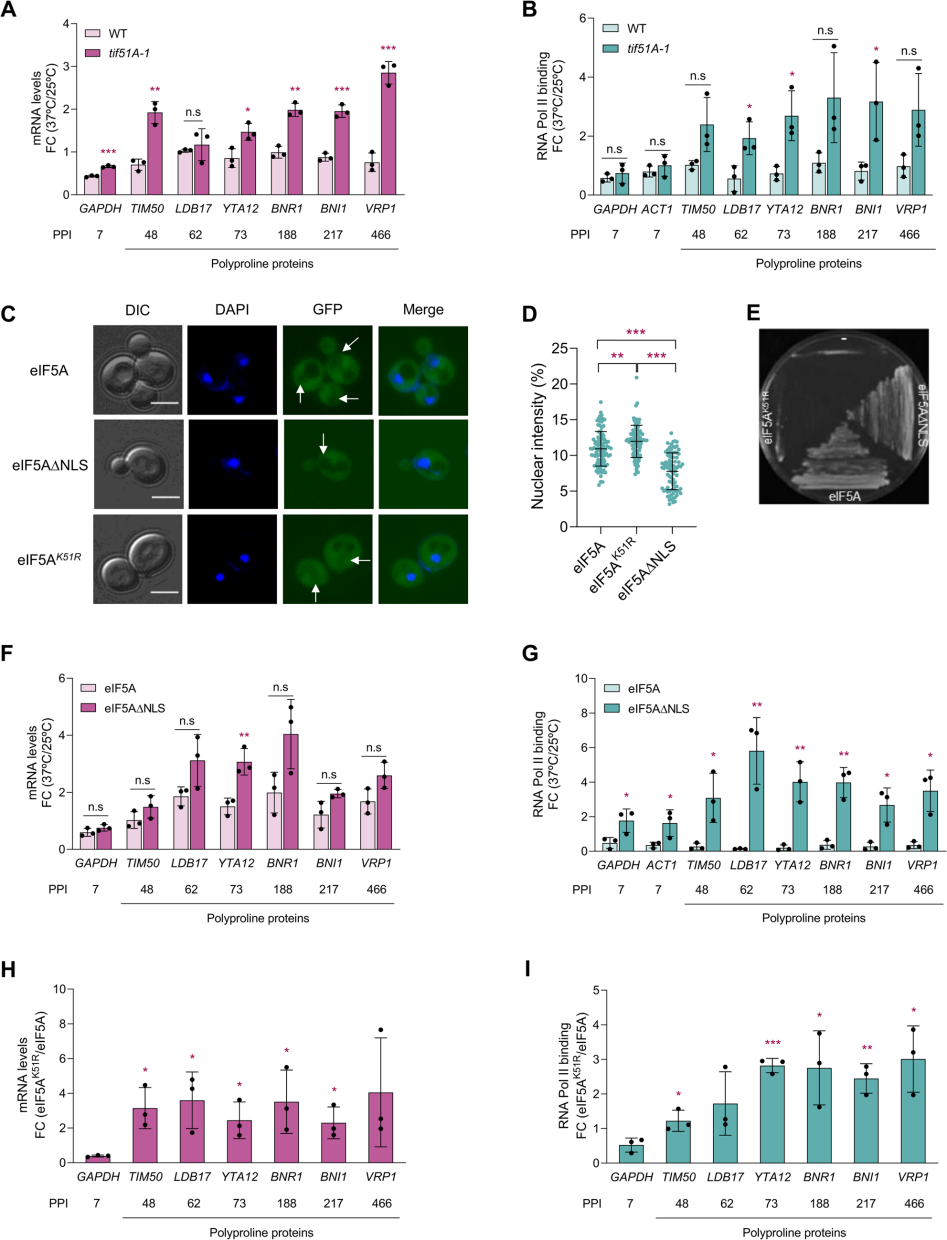
eIF5A depletion, nuclear exclusion, or deficient hypusination increases the transcription of eIF5A translation-dependent genes. **A**,**B** Wild-type and *tif51A-1* mutant yeast strains were grown in YPD medium at 25ºC until exponential phase and then transferred to 25ºC and 37ºC for four hours. **A** mRNA levels from each gene were determined by RT-qPCR using primers designed for amplification in the ORF regions. **B** ChIP of RNA Pol II was performed using the antibody anti-Rpb1 (8WG16). The immunoprecipitated DNA was used to quantify the binding to the different genes by qPCR using primers designed for amplification in the ORF regions. The percentage of the signal obtained in each ChIP sample with respect to the signal obtained with the DNA from the corresponding whole cell extract was calculated. **C** Wild-type strain expressing a second copy of GFP-eIF5A, GFP-eIF5A∆NLS or GFP-eIF5A^*K51R*^ were cultured in YPD medium until reaching exponential phase and subjected to fluorescence microscopy. Cells were incubated for 5 min with DAPI prior microscopy to stain the nuclei. White arrows indicate the nuclei. A representative image is shown from three independent experiments. Scale bar, 4 μm. **D** Quantification of percentage of nuclear signal is shown from a minimum of 100 cells. Results are presented as individual values together with the mean ± SD from three independent experiments. The statistical significance was determined by using a Kruskal–Wallis test followed by Wilcoxon rank-sum analysis. **E** *tif51A-1* strain with or without (-) the expression of a second copy GFP-eIF5A, GFP-eIF5A∆NLS or GFP-eIF5A^*K51R*^ was cultured in YPD at 37ºC to deplete eIF5A from the first copy (Tif51A-1 protein). **F**,**G** Wild-type expressing GFP-eIF5A or GFP-eIF5A∆NLS in the *TIF51A* locus were grown as in (**A**) and mRNA levels (**F**) and RNA Pol II ChIP binding (**G**) were measured as in (**A**,**B**). **A**,**B**,**F**,**G** Data are presented as the mean fold change 37ºC vs. 25ºC ± SD from three independent experiments. The statistical significance was determined by using a Kruskal–Wallis followed by Wilcoxon rank-sum analysis. **H**,**I** Yeast strain with tetO_7_-*TIF51A* at the endogenous locus and a second copy of GFP-eIF5A or GFP-eIF5A^*K51R*^ were cultured overnight in YPD at 25ºC in the presence of doxycycline (15 µM) to deplete eIF5A from the first copy, and then mRNA levels (**H**) and RNA Pol II ChIP binding (**I**) were measured as in (**A**,**B**). Data are presented as the mean fold change eIF5A^*K51R*^ vs. eIF5A wild-type ± SD from three independent experiments. Statistical significance was determined using a two-tailed unpaired Student’s t-test relative to wild-type cells. For all experiments *p < 0.05, **p < 0.01, ***p < 0.001. n.s means no significant differences

To validate our genome-wide data, we measured the changes in mRNA levels (RA) of several specific genes showing differences in their eIF5A translation-dependencies, according to their PPI values. Results showed a statistically significant increase in mRNA levels in eIF5A-depleted *tif51A-1* cells with respect to the wild-type for most of the tested genes (Fig. 3A). Consistent with our GRO results, this increase correlated well with the PPI values, indicating that the stronger the eIF5A translation dependency, the higher the mRNA abundance.

To independently corroborate that the increase in mRNA levels of eIF5A-translation target genes upon eIF5A depletion is due to an increase in SR (Fig. 2D-F), we investigated the occupancy of total RNA Pol II, as a proxy of transcriptional activity. We performed chromatin immunoprecipitation (ChIP) using an antibody against the catalytical Rpb1 subunit of RNA Pol II and observed that total RNA Pol II was recruited to similar levels in wild-type and *tif51A-1* cells to eIF5A translation-independent genes, such as *ACT1* or *GAPDH*. However, and consistent to our GRO results, we found a higher and significant association (2- to fourfold increase) of the total RNA Pol II to the ORFs of genes showing a strong eIF5A-dependence for their translation in the *tif51A-1* cells (Fig. 3B). The higher binding of the RNA Pol II elongation complex was in accordance to the higher mRNA levels of the affected genes (Fig. 3A).

We next investigated whether the transcriptional derepression observed upon eIF5A depletion was linked to a nuclear function of eIF5A. To address this, we first identified a nuclear localization signal (NLS) in the eIF5A yeast protein in order to split nuclear and cytoplasmic eIF5A functions. In mammalian cells it has been proposed that the eIF5A N-terminal protein region acts as NLS, although bearing no structural similarity with classical nuclear localization signals [45]. In *S. cerevisiae*, the N-terminal (1-MSDEEHTFETADAGSSATY-19) extension of the eIF5A protein is enriched in acidic residues and conserved across eukaryotes but absent in bacteria and archaea orthologues (Supplementary Figure S4A) and, therefore, candidate to act as NLS. We constructed yeast strains expressing an additional copy of full eIF5A sequence or a ΔNLS version of eIF5A (Δ1-19) fused to GFP. Importantly, both GFP-tagged eIF5A versions demonstrated to be functional (Fig. 3E). Direct *in vivo* visualization of GFP-eIF5A revealed a whole cell distribution pattern with a primary cytoplasmic signal, consistent with its cytoplasmic function, and a faint nuclear signal corresponding to approximately 12% of the total signal (Fig. 3C,D). This partial nuclear localization of eIF5A under standard conditions aligns well with previous observations in mammalian cells [14, 17–19, 45, 46] and confirms the shuttling of eIF5A between nucleus and cytoplasm. However, deletion of the N-terminal region in yeast eIF5A (GFP-eIF5AΔNLS) hindered its nuclear import (Fig. 3C,D). This demonstrated that the N-terminal NLS is necessary for signaling eIF5A nuclear accumulation in *S. cerevisiae* cells. Conversely, deletion of Pdr6, the described nuclear exportin of eIF5A [15, 16], resulted in increased nuclear accumulation of GFP-eIF5A (Supplementary Figure S4B,C). Interestingly, the nuclear localization of eIF5A was found to be specific and clearly different from that of other translation factors such as eIF4E, which is clearly excluded from the nucleus under standard conditions (Supplementary Figure S4B,C).

Because both GFP-eIF5A and GFP-eIF5AΔNLS expressed as a second copy complemented growth of *tif51A-1* mutant at 37 °C (Fig. 3E) and GFP-eIF5AΔNLS was activated by hypusination (Supplementary Figures S4D,E), we generated new strains with either of the two eIF5A versions at its natural chromosomal locus and confirmed their viability (Supplementary Figure S4F). As expected, blocking the nuclear import of eIF5A compromised its association to the chromatin (Supplementary Figure S4G). Then, we examined the changes in mRNA levels of genes with different eIF5A translation-dependencies (Fig. 3F) and found that cells with the eIF5A∆NLS version exhibited an increase in mRNA levels compared to wild-type cells, and that this increase correlated with the PPI values (Fig. 3F). In parallel, we tested the association of RNA Pol II by ChIP and observed a significantly higher recruitment (3- to fivefold increase) to the ORFs of genes with the highest PPI values (Fig. 3G). Importantly, changes in both mRNA levels and RNA Pol II binding in eIF5A∆NLS cells were similar to those in *tif51A-1* cells.

We then explored whether inhibiting the translational activity of eIF5A might affect its nuclear function in gene repression. To achieve this, we used an eIF5A version with a hypusine site mutation (*K51R*), which cannot be modified into the hypusine form [47]. First, we confirmed that the mutation impaired hypusination (Supplementary Figures S4D,E) and induced eIF5A nuclear accumulation and loss of protein function (Fig. 3C,D,E), consistent with previous reports [17, 18]. Next, we constructed a yeast strain in which the *TIF51A* gene (expressing eIF5A), was under the control of a tetO_7_ promoter and confirmed that switching off the native eIF5A gene by adding doxycycline resulted in lack of growth and increased mRNA levels of the eIF5A translation-target genes, as it happens with the *tif51A-1* temperature-sensitive mutant at restrictive temperature (Supplementary Figure S4H,I). Then, we engineered this conditional yeast strain to add a second copy of either GFP-eIF5A^*K51R*^ or a control GFP-eIF5A version. After adding doxycycline to the culture, the eIF5A from the native locus is depleted, meaning that the cells only express the GFP fusions with the wild-type or *K51R* versions of eIF5A. As expected, both the GFP-eIF5A^*K51R*^ and the GFP-eIF5A proteins were expressed, but only the second version was hypusinated and rescued growth after the addition of doxycycline (Supplementary Figure S4I,J,K). After doxycycline addition, we observed that both the mRNA levels and RNA Pol II recruitment increased for the genes encoding translational targets of eIF5A in cells expressing the eIF5A^*K51R*^ mutant protein with respect to the ones expressing GFP-eIF5A (Fig. 3H,I). We also observed a slight but consistent increase in GFP-eIF5A^*K51R*^ protein levels when the native eIF5A protein was down-regulated by the addition of doxycycline (see Supplementary Figure S4K). Therefore, we cannot completely rule out the possibility that this increase has had an effect. However, the changes in both mRNA levels and RNA Pol II binding in eIF5A^*K51R*^-expressing cells were similar to those in *tif51A-1* cells, suggesting that the absence of eIF5A or of a cytoplasmic functional eIF5A dysregulates transcription.

Taken together, these results suggest that the repression of transcription by eIF5A requires its nuclear localization, as this is abolished when its NLS is removed. Moreover, it also requires a functional eIF5A for cytoplasmic translation, since cells that only express a non-hypusinated eIF5A protein, which cannot bind ribosomes, do not show the transcriptional repression of eIF5A translation-targets, despite presenting a higher nuclear accumulation of the eIF5A^*K51R*^ protein. Therefore, these results suggest that nuclear eIF5A plays a direct role in transcriptional repression and that this is functionally linked to its activity in translation.

### The presence of polyproline motifs in eIF5A translation-dependent genes is sufficient to promote their repression by eIF5A

Our discovery that eIF5A preferentially binds to the chromatin of genes that encode target proteins for translation –a process that is predominantly RNA-mediated- and its correlation to the transcriptional regulation by eIF5A, prompted us to investigate the chromatin/RNA feature that mediates eIF5A specificity. Several of the strongest identified eIF5A translation-targets contain polyproline motifs, resulting in high PPI values [10, 11]. We explored the effect in transcription of removing these motifs from eIF5A translation targets and of adding these motifs to translation-independent genes.

**Fig. 4:**
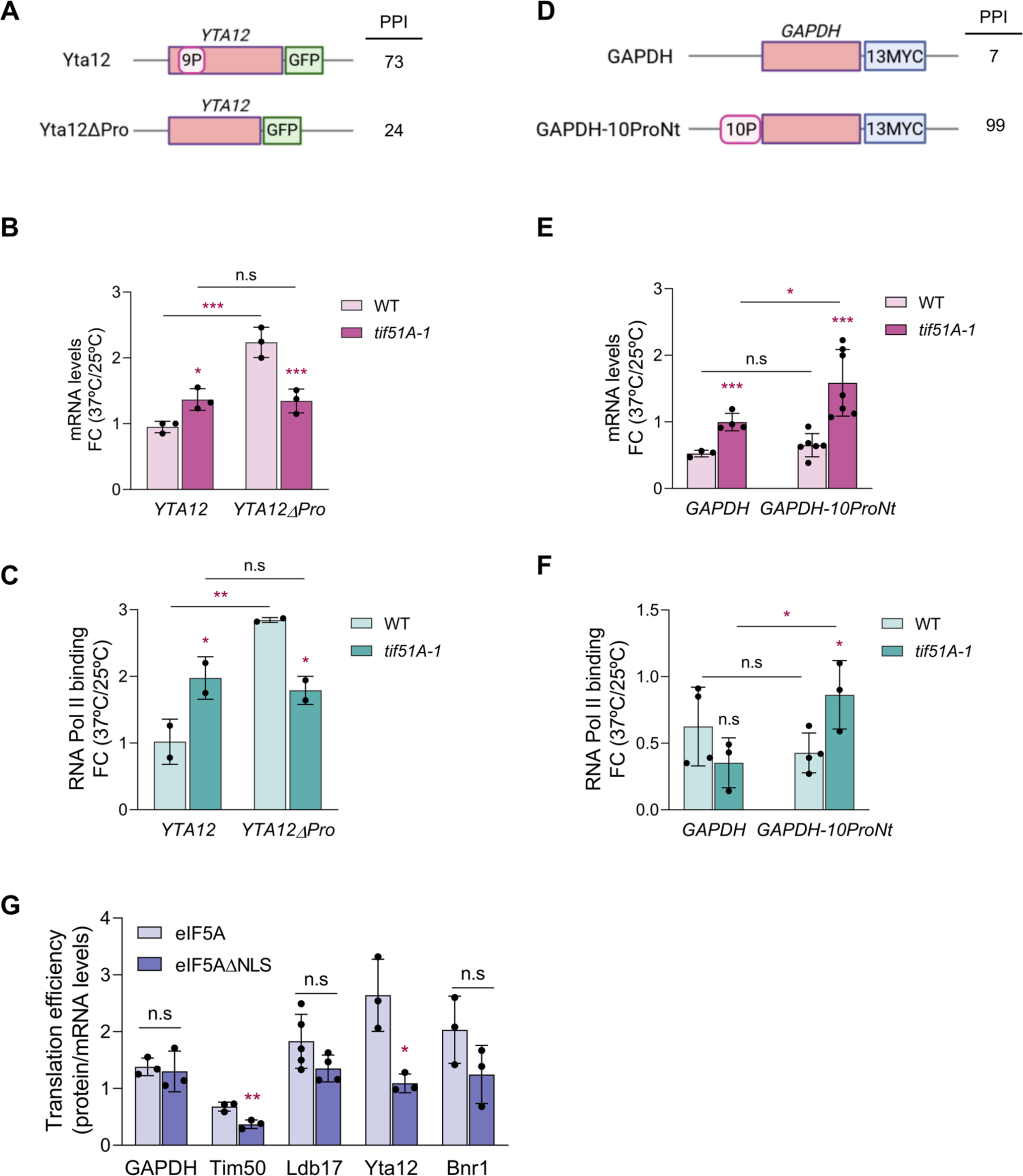
Transcriptional repression driven by polyPro encoding sequences promotes the translation efficiency of eIF5A target proteins. **A**,**D** Schematic diagram showing the C-terminal genomic tagging of native *YTA12* (**A**) and *GAPDH* (**D**) ORFs as well as their polyPro-deleted (A, *YTA12ΔPro*) or -inserted (**D**, *GAPDH*-10ProNt) versions. PPI of all gene versions are shown. **B**,**E** Wild-type and *tif51A-1* mutant yeast strains carrying the native and the mutated versions of *YTA12* and *GAPDH* genes were grown in YPD medium at 25ºC until exponential phase and then transferred to 25ºC and 37ºC for four hours. mRNA levels from *YTA12* (**B**) and *GAPDH* (**E**) versions were determined by RT-qPCR. **C**,**F** Wild-type and *tif51A-1* mutant yeast strains carrying the native and the mutated versions were grown as in (**B**,**E**). ChIP of RNA Pol II was performed using the antibody anti-Rpb1 (8WG16). The immunoprecipitated DNA was used to quantify the binding to *YTA12* (**C**) and *GAPDH* (**F**) genes by qPCR using primers designed for amplification in the specific ORF regions. The percentage of the signal obtained in each ChIP sample with respect to the signal obtained with the DNA from the corresponding whole cell extract was calculated. **B**,**C**,**E**,**F** Data are presented as the mean ± SD from at least three independent experiments. Statistical significance was determined by using a two-way ANOVA followed by Estimated Marginal Means (emmeans) post-hoc tests. **G** Wild-type cells expressing the native eIF5A or the eIF5A∆NLS version were grown in YPD medium at 30ºC until exponential phase. mRNA and protein levels from *GAPDH*, *TIM50*, *LDB17*, *YTA12* and *BNR1* genes were determined and quantified (see Supplemental Figure S5), and then translation efficiency was determined as the ratio between protein and mRNA levels. Data are presented as the mean ± SD from at least three independent experiments. Statistical significance was determined using a two-tailed unpaired Student’s t-test relative to corresponding wild-type cells. For all experiments *p < 0.05, **p < 0.01, ***p < 0.001. n.s means no significant differences

Following this idea, we first obtained yeast strains targeting the genes *YTA12*, encoding a proline-rich component of the mitochondrial m-AAA protease, *TIM50* and *BNR1*, which have high PPI values and have been previously shown to be bona-fide translation targets of eIF5A [39, 40, 48]. Here, we have shown that these genes present high levels of eIF5A binding (Fig. 1G), and that respond to nuclear eIF5A depletion by increasing RNA Pol II binding and mRNA levels (Fig. 3A,B,F,G). For each gene, we obtained two genomic GFP- or HA-tagged versions in wild-type and *tif51A-1* cells: one with GFP or HA after the native coding region (*YTA12*-GFP, *BNR1*-HA and *TIM50*-GFP) and a mutated version in which the region containing the polyPro stretches was deleted (*YTA12∆Pro*-GFP, *BNR1∆Pro*-HA and *TIM50∆Pro*-GFP). These new versions, therefore, showed lower PPI values and were predicted to behave as eIF5A translation-independent genes (Fig. 4A and Supplementary Figs. 5A,B; [39].

We analyzed the mRNA levels in the new strains and found similar results for the three genes. We found a significant increase in the mRNA levels of the three native genes (expressing the polyPro motifs) in the *tif51A-1* cells following the temperature shift. However, we found that this mRNA increase was no longer present under eIF5A depletion for the mutated versions of the three genes without the polyPro motifs (Fig. 4B,C and Supplementary Figure S5C,D). Moreover, we observed that the polyPro sequences had a repressive effect in the presence of eIF5A (wild-type strain), as the mRNA levels were lower for the *YTA12*, *BNR1* and *TIM50* genes carrying the polyPro motifs than for the ∆Pro protein versions (Fig. 4B and Supplementary Figure S5C,D). Next, we evaluated the recruitment of the RNA Pol II to the *YTA12* gene by performing ChIP analysis of RNA Pol II. We found that polymerase binding to the *YTA12* gene body was higher upon eIF5A depletion in the *tif51A-1* cells, but only for the polyPro-containing *YTA12*-GFP gene and not for the YTA12∆Pro-GFP gene without the proline stretches (Fig. 4C).

Next, we aimed to test the impact on mRNA levels and transcription following the insertion of a polyPro region into an eIF5A-translation independent gene. For this, we generated wild-type and *tif51A-1* strains targeting the *GAPDH* gene (isoform *TDH1*), that encodes the glyceraldehyde-3-phosphate dehydrogenase enzyme involved in glycolysis and gluconeogenesis. We generated genomic C-terminal Myc-tagged strains: one with the native coding region (*GAPDH*-myc) and low PPI value, and a mutated version in which 10 consecutive prolines inserted at the N-terminal region after the first codon (GAPDH-10ProNt-myc). As expected, only the version with polyPro motifs (high PPI) behaved as an eIF5A-dependent gene for translation (Fig. 4D and Supplementary Figure S5E,F). Using these new strains, we observed a slight but significant increase in the mRNA levels of the native gene (*GAPDH*-myc) in the *tif51A-1* cells after the temperature shift. However, we found a significantly greater increase in the mRNA levels of the *GAPDH*-10ProNt-myc version in the *tif51A-1* cells (Fig. 4E). We also observed that the association of the RNA Pol II to the *GAPDH* gene body was similar across strains carrying the *GAPDH*-myc whereas its recruitment was significantly higher in *tif51A-1* cells carrying the GAPDH-10ProNt-myc version compared to the wild-type (Fig. 4F).

In summary, these results suggest that the presence of polyPro motifs, which stall translation under eIF5A deficiency, are both necessary and sufficient to drive the eIF5A-dependent transcriptional regulation.

### The coordinated nuclear and cytoplasmic functions of eIF5A facilitate the efficient synthesis of its target proteins

We were prompted to investigate the biological advantages of this double but opposite eIF5A-dependent gene regulation, as the results suggested that nuclear eIF5A drives transcriptional repression while promoting the cytoplasmic translation of the same targets. To do this, we used the eIF5A∆NLS version, which is mostly excluded from the nucleus (Fig. 3C,D), does not repress transcription (Fig. 3F,G) but retains its translation function, thereby maintaining cellular viability (Fig. 3E). We then compared this ∆NLS version with the native eIF5A version, which is active in both the nucleus and the cytoplasm. As previously demonstrated, both eIF5A∆NLS and eIF5A strains grew well in glucose media at 30 °C and had similar doubling times (Supplementary Figure S6C); therefore, this result did not indicate superior performance of native eIF5A under standard conditions. We then investigated whether translation efficiency differed in yeast cells carrying either of the eIF5A versions. To do this, we measured the mRNA and protein levels of several eIF5A translation-dependent genes (*TIM50*, *LDB17*, *YTA12* and *BNR1*), as well as a control gene (*GAPDH*), and then calculated the protein/mRNA ratio as an indicator of translation efficiency. The results again showed that inhibiting the nuclear-cytoplasmic shuttling of eIF5A increased the mRNA levels of target genes (Supplementary Figure S6D); however, we did not observe an increase in the corresponding protein levels (Supplementary Figure S6E,F). Consequently, estimation of the protein-to-mRNA ratios revealed lower translation efficiencies for eIF5A-dependent genes when nuclear localization of eIF5A was inhibited (Fig. 4G), which was not due to lower eIF5A∆NLS expression (Supplementary Figure S6A,B). Thus, these results suggest that reducing transcription by nuclear eIF5A positively affects the subsequent eIF5A-dependent cytoplasmic translation of the corresponding target mRNA. However, this slight difference in the translation efficiency of target genes does not result in improved growth under normal conditions.

## Discussion

In recent years, the crosstalk between mRNA synthesis, maturation, nuclear export and decay has been established [29]. However, there is less information on the interaction between two of the most physically and temporally separated processes: transcription and translation.

Here, we found that the highly abundant translation elongation factor eIF5A associates with the chromatin of many genes in yeast cells. However, we identified a group of genes with higher binding of eIF5A where eIF5A appears to act as a repressor, indicated by a relative increase in transcription, RNA Pol II binding and the corresponding mRNA levels upon eIF5A depletion, and showing a different pattern to global regulation. This group of genes mainly encodes proteins with high levels of tripeptide motifs (e.g. polyPro), which have been identified as conferring dependency on eIF5A for translation of the corresponding mRNA [10, 11]. While there is currently no experimental evidence for the dependency on eIF5A for the translation of all genes in this group with high PPI, we have shown for several of them, which are bona fide eIF5A translation targets conserved in humans (e.g. *TIM50*, *YTA12*, *BNI1*, *BNR1*, *VRP1*), that are bound by eIF5A, and that their RNA Pol II association and mRNA levels depend on the presence of nuclear eIF5A. These results strongly suggest a direct role for eIF5A in negatively regulating their transcription.

Our results point that transcription is regulated by the presence of eIF5A in the nucleus, since the regulation is lost when using a version of eIF5A without the here identified nuclear localization signal. However, the precise mechanism involved still remains unknown. Previous data have shown that eIF5A interacts with RNA Pol II in precursor neurons [25], and that under hypoxia the mammalian eIF5A2 isoform binds to the HIF1-1a gene where it may positively regulate its transcription [26, 27]. The presence of a conserved NLS in the N-terminal extension of the eukaryotic eIF5A proteins suggests that nuclear localization of the protein has a relevant function that has been conserved through evolution. Beyond its nuclear localization, this transcriptional regulation also requires the active engagement of cytosolic eIF5A in translation, as it is lost in cells expressing the eIF5A^*K51R*^ mutant, which cannot undergo hypusination and therefore fails to bind ribosomes and regulate translation but still shuttles to the nucleus. This result suggests a link between the two activities of the protein. However, we cannot rule out the possibility that the *K51R* mutation produces a structural modification to the eIF5A protein, which may inhibit its nuclear activity in the regulation of transcription.

Furthermore, our results point to eIF5A's association with chromatin relying on the presence of RNA. This suggests that, like other RBP proteins, eIF5A may bind to nascent RNA co-transcriptionally to modulate transcription. eIF5A structural features suggest a potential to interact with nucleic acids. The C-terminal domain resembles the cold-shock domain, common in DNA and RNA-binding proteins, while the N-terminal carries the hypusine residue, which contains two positive charges and resembles spermidine, a molecule known to interact with DNA or RNA [49, 50]. In this line, eIF5A has been previously described to interact directly or indirectly with mRNAs [21, 51]. For instance, it functions as a co-factor of the HIV-1 Rev protein, thereby modulating transcription, translation, and replication of the viral genome in mammalian cells [20, 22]. Beyond viral infection, eIF5A has been linked to mRNA export, including *NOS2* in a diabetic inflammation model [23] and *TSC2* under anaerobic stress [24]. Recently, eIF5A was identified as an RNA-binding protein in a time-resolved profiling study in mammalian cells, where it was unexpectedly captured bound to mRNAs at early stage points of mRNA life cycle and before nuclear export [52].

As the binding of eIF5A to nascent mRNA seems plausible, how it specifically recognizes its target mRNA is a primordial question. We found that nucleotide sequences encoding polyPro motifs confer dependency on eIF5A not only for translation but also for transcriptional regulation. Codons encoding prolines (CCX sequence) create C-rich RNA regions that could form a recognition motif. Similarly, other eIF5A-dependent tripeptide motifs contributing to PPI also contain proline in their sequences [10, 11]. In this sense, the presence of specific C-rich motifs in mRNAs has been shown to serve as platform for binding different RBPs [53, 54]. Further research is needed to clarify this issue.

**Fig. 5:**
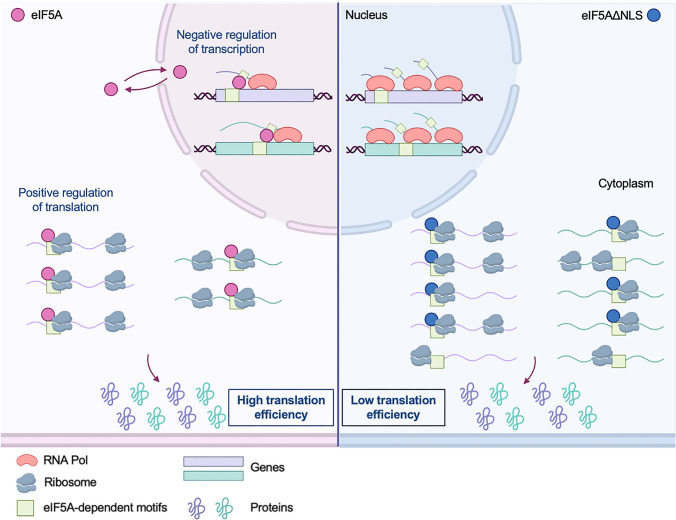
Model showing the proposed role of eIF5A in the regulation of transcription. The eukaryotic translation factor eIF5A helps cytosolic translation elongation of proteins with eIF5A-dependent motifs in their sequences and rescues ribosome stalling. eIF5A is also present in the nucleus, most likely in an acetylated and non-hypusinated form, where it binds to eIF5A-dependent genes and down-regulates their transcription, thereby tightly controlling mRNA synthesis and, consequently, final protein production (left). Restricting eIF5A to the cytoplasm (eIF5AΔNLS), triggers increased relative transcription of eIF5A-dependent genes in a context of decreased global transcription. However, the resulting higher levels of mRNA for eIF5A-dependent genes do not lead to increased production of proteins, as the availability of eIF5A in the cytoplasm may become limiting due to the increased levels of target mRNA, despite the small increase in cytoplasmic eIF5A (right). Consequently, the translation efficiency (measured as the protein-to-mRNA ratio) is lower with eIF5AΔNLS than with wild-type eIF5A present both at the nucleus and the cytoplasm

Our results raise another relevant question: what are the biological benefits of regulating translation and transcription by the same protein, thus yielding coordinated regulation? We demonstrated that the translation efficiency of target genes is reduced when eIF5A is predominantly excluded from the nucleus. Thus, inhibiting the transcriptional repression of eIF5A translation-target genes using eIF5AΔNLS increased mRNA levels, but not the levels of the corresponding proteins. These results suggest that translation efficiency improves when eIF5A associates with chromatin early on. In this context, it has been described that the highly abundant translation elongation factor 1A (eEF1A), essential for delivering aminoacyl-tRNA to the ribosome for translation, also shuttles between the nucleus and the cytoplasm, and binds to the chaperone mRNA encoding Hsp70 to couple its transcription and translation during heat stress (HS) in mammalian cells [55]. Altogether, results suggest the possibility of nascent mRNA imprinting by translation elongation factors that shuttle between the nucleus and the cytoplasm, facilitating the subsequent synthesis of encoded proteins. In the case of eIF5A, however, transcription and translation are regulated in opposite directions: transcription is repressed and translation is promoted. We propose that it may be beneficial for the cell to synthesize fewer eIF5A target mRNAs, as the capture of too much cellular eIF5A protein by those mRNAs could deplete its cytoplasmic pool and interfere with other essential functions (see Model in Fig. 5). In this respect, recent work has shown that the aggregates of the pathological Huntingtin (mHTT) protein traps eIF5A protein in the brains of mice, and has proposed that this eIF5A depletion disrupts homeostatic control and impairs cellular recovery from stress [56]. Additionally, it has recently been proposed that eIF5A could play a role in global translation progression and connect ribosomal progress with quality surveillance [57]. Unresolved ribosome stalling in mRNAs due to eIF5A protein scarcity could lead to intense competition for ribosomes, which is the main factor influencing translation costs and, consequently, cellular fitness [58]. It is worth noting that the nucleus-cytoplasm shuttling of eIF5A is regulated by post-translational modifications of the protein, such as hypusination, sumoylation, and acetylation [17–19], which may respond to signaling cues activated by changing conditions. In this line, several studies in mammals have described the translocation of eIF5A to the nucleus in response to hypoxia and apoptosis induction [26, 59]. Future systematic studies under different conditions may reveal the beneficial effects of transcription-translation crosstalk by eIF5A. Furthermore, given that some of the eIF5A transcriptional targets identified in yeast have been associated with human diseases (e.g. formins, Tim50, Yta12 and Vrp1), it could be of therapeutical interest to study if the eIF5A-mediated transcriptional regulation is conserved across higher eukaryotes.

## Supplementary Information

Below is the link to the electronic supplementary material.

Supplementary file1 (PDF 1270 KB)

## Data Availability

ChIP-seq data is deposited in NCBI’s Gene Expression Omnibus (GEO) under accession number GSE303994. The GRO data is deposited in GEO under accession number GSE302334.
