# Supplementary material for: eIF5A coordinates the transcription and translation of its target genes
Source: Cell Mol Life Sci. 2026 May 22;83(1):282. doi: 10.1007/s00018-026-06252-8 (PMC13376067; doi:10.1007/s00018-026-06252-8)
Supplement: Supplementary file 1 — Supplementary file1 (PDF 1270 KB) [file 18_2026_6252_MOESM1_ESM.pdf]

Supplementary information (SI)

eIF5A coordinates the transcription and translation of its target genes

Cellular and Molecular Life Sciences

Marina Barba-Aliaga[1,2,5]*, Lianqi Chi[1,2], Samoa Prieto-Díez[1,2], Jordi Planells[1,3], José García-Martínez[1,4], José E. Pérez-Ortín[1,2]* and Paula Alepuz[1,2]*

[1] Instituto de Biotecnología y Biomedicina (Biotecmed), Universitat de València, 46100 València, Spain
[2] Departamento de Bioquímica y Biología Molecular, Universitat de València, 46100 València, Spain
[3] Departamento de Biología Celular, Biología Funcional y Antropología Física, Universitat de València, 46100 València, Spain
[4] Departamento de Genética, Universitat de València, 46100 València, Spain
[5] Current address: Department of Molecular and Cellular Biology, Faculty of Arts and Sciences, Harvard University, Cambridge, MA 02138, USA

*Corresponding authors: Paula Alepuz (https://orcid.org/0000-0003-1472-2373), Marina Barba-Aliaga (https://orcid.org/0000-0002-1842-9126), José E. Pérez-Ortín (https://orcid.org/0000-0002-1992-513X)

Email: paula.alepuz@uv.es; mbarbaaliaga@fas.harvard.edu; jose.e.perez@uv.es

Table of contents:

1. **Supplementary Tables S1-S4**
2. **Supplementary Materials and methods**
3. **Supplementary Figures S1-S6**

1. Supplementary Tables

Supplementary Table S1. Yeast strains used in this study

Name	Genotype	Source
**BY4741**	MATa *ura3Δ0 leu2Δ0 his3Δ1 met15Δ0*	Euroscarf
tif51A-1	BY4741 MATa *ura3Δ0 leu2Δ0 his3Δ1 met15Δ0 tif51A-1::kanR*	[1]
**PAY666**	BY4741 MATa *ura3Δ0 leu2Δ0 his3Δ1 met15Δ0 eIF4e-GFP-his3MX6*	This study
**PAY778**	BY4741 MATa ura3Δ0 leu2Δ0 his3Δ1 met15Δ0 *tetO$_7$-TIF51A-kanMX*	This study
**PAY888**	BY4741 MATa *ura3Δ0 leu2Δ0 his3Δ1 met15Δ0 TIM50-GFP-his3MX6*	[2]
**PAY890**	BY4741 MATa *ura3Δ0 leu2Δ0 his3Δ1 met15Δ0 tif51A-1::kanR TIM50-GFP-his3MX6*	[2]
**PAY892**	BY4741 MATa *ura3Δ0 leu2Δ0 his3Δ1 met15Δ0 YTA12-GFP-his3MX6*	[2]
**PAY893**	BY4741 MATa *ura3Δ0 leu2Δ0 his3Δ1 met15Δ0 tif51A-1::kanR YTA12-GFP-his3MX6*	[2]
**PAY896**	BY4741 MATa *ura3Δ0 leu2Δ0 his3Δ1 met15Δ0 BNR1-3HA-his3MX6*	This study
**PAY898**	BY4741 MATa *ura3Δ0 leu2Δ0 his3Δ1 met15Δ0 tif51A-1::kanR BNR1-3HA-his3MX6*	This study
**PAY899**	BY4741 MATa *ura3Δ0 leu2Δ0 his3Δ1 met15Δ0 BNR1ΔPro-3HA-his3MX6*	This study
**PAY901**	BY4741 MATa *ura3Δ0 leu2Δ0 his3Δ1 met15Δ0 tif51A-1::kanR BNR1ΔPro-3HA-his3MX6*	This study
**PAY1083**	BY4741 MATa *ura3Δ0 leu2Δ0 his3Δ1 met15Δ0 GFP-TIF51A-his3MX6*	This study
**PAY1085**	BY4741 MATa *ura3Δ0 leu2Δ0 his3Δ1 met15Δ0 TIM50ΔPro-GFP-his3MX6*	[2]
**PAY1086**	BY4741 MATa *ura3Δ0 leu2Δ0 his3Δ1 met15Δ0 tif51A-1::kanR TIM50ΔPro-GFP-his3MX6*	[2]
**PAY1087**	BY4741 MATa *ura3Δ0 leu2Δ0 his3Δ1 met15Δ0 YTA12ΔPro-GFP-his3MX6*	This study
**PAY1088**	BY4741 MATa *ura3Δ0 leu2Δ0 his3Δ1 met15Δ0 tif51A-1::kanR YTA12ΔPro-GFP-his3MX6*	This study
**PAY1443**	BY4741 MATa *ura3Δ0 leu2Δ0 his3Δ1 met15Δ0 GAPDH-13myc-his3MX6*	This study
**PAY1444**	BY4741 MATa *ura3Δ0 leu2Δ0 his3Δ1 met15Δ0 tif51A-1::kanR GAPDH-13myc-his3MX6*	This study
**PAY1631**	BY4741 MATa *ura3Δ0 leu2Δ0 his3Δ1 met15Δ0 GFP-TIF51A^{K51R}-his3MX6*	This study
**PAY1632**	BY4741 MATa *ura3Δ0 leu2Δ0 his3Δ1 met15Δ0 GFP-TIF51AΔNLS-his3MX6*	This study
**PAY1633**	BY4741 MATa *ura3Δ0 leu2Δ0 his3Δ1 met15Δ0 pdr6::kanMX6 GFP-TIF51A-his3MX6*	This study
**PAY1673**	BY4741 MATa *ura3Δ0 leu2Δ0 his3Δ1 met15Δ0 tif51A-1::kanR GFP-TIF51A-his3MX6*	This study
**PAY1675**	BY4741 MATa *ura3Δ0 leu2Δ0 his3Δ1 met15Δ0 tif51A-1::kanR GFP-TIF51A^{K51R}-his3MX6*	This study
**PAY1678**	BY4741 MATa *ura3Δ0 leu2Δ0 his3Δ1 met15Δ0 tif51A-1::kanR GFP-TIF51AΔNLS-his3MX6*	This study
**PAY1759**	BY4741 MATa *ura3Δ0 leu2Δ0 his3Δ1 met15Δ0 tif51A::GFP-TIF51A-his3MX6*	This study
**PAY1760**	BY4741 MATa *ura3Δ0 leu2Δ0 his3Δ1 met15Δ0 tif51A::GFP-TIF51AΔNLS-his3MX6*	This study
**PAY1915**	BY4741 MATa *ura3Δ0 leu2Δ0 his3Δ1 met15Δ0 tif51A::GFP-TIF51A-his3MX6 LDB17-13myc-KanMX*	This study
**PAY1916**	BY4741 MATa *ura3Δ0 leu2Δ0 his3Δ1 met15Δ0 tif51A::GFP-TIF51AΔNLS-his3MX6 LDB17-13myc-KanMX*	This study
**PAY1917**	BY4741 MATa *ura3Δ0 leu2Δ0 his3Δ1 met15Δ0 tif51A::GFP-TIF51A-his3MX6 BNR1-3HA-KanMX*	This study
**PAY1919**	BY4741 MATa *ura3Δ0 leu2Δ0 his3Δ1 met15Δ0 tif51A::GFP-TIF51AΔNLS-his3MX6 BNR1-3HA-KanMX*	This study
**PAY1927**	BY4741 MATa *ura3Δ0 leu2Δ0 his3Δ1 met15Δ0 tif51A::GFP-TIF51A-his3MX6 GAPDH-13myc-KanMX*	This study
**PAY1928**	BY4741 MATa *ura3Δ0 leu2Δ0 his3Δ1 met15Δ0 tif51A::GFP-TIF51AΔNLS-his3MX6 GAPDH-13myc-KanMX*	This study
**PAY1948**	BY4741 MATa *ura3Δ0 leu2Δ0 his3Δ1 met15Δ0 tif51A::GFP-TIF51A-his3MX6 YTA12-GFP-KanMX*	This study
**PAY1949**	BY4741 MATa *ura3Δ0 leu2Δ0 his3Δ1 met15Δ0 tif51A::GFP-TIF51AΔNLS-his3MX6 YTA12-GFP-KanMX*	This study
**PAY1950**	BY4741 MATa *ura3Δ0 leu2Δ0 his3Δ1 met15Δ0 tif51A::GFP-TIF51A-his3MX6 TIM50-3HA-KanMX*	This study

**PAY1952**	BY4741 MATa *ura3Δ0 leu2Δ0 his3Δ1 met15Δ0 tif51A::GFP-TIF51AΔNLS-his3MX6 TIM50-3HA-KanMX*	This study
**PAY2118**	BY4741 MATa *ura3Δ0 leu2Δ0 his3Δ1 met15Δ0 tetO$_7$-TIF51A-kanMX GFP-TIF51A-his3MX6*	This study
**PAY2130**	BY4741 MATa *ura3Δ0 leu2Δ0 his3Δ1 met15Δ0 GAPDH-10ProNt-13myc-his3MX6*	This study
**PAY2132**	BY4741 MATa *ura3Δ0 leu2Δ0 his3Δ1 met15Δ0 tif51A-1::kanR GAPDH-10ProNt-13myc-his3MX6*	This study
**PAY2158**	BY4741 MATa *ura3Δ0 leu2Δ0 his3Δ1 met15Δ0 tetO$_7$-TIF51A-kanMX GFP-TIF51A^{K51R}-his3MX6*	This study
**PAY2246**	BY4741 MATa *ura3Δ0 leu2Δ0 his3Δ1 met15Δ0 CTK1-GFP-his3MX6*	This study

Supplementary Table S2. Plasmids used in this study

Name	Plasmid description	Source
**PA201**	pFA6a-3HA-HIS3MX6	[3]
**PA234**	pFA6a-13myc-HIS3MX6	[3]
**PA235**	pFA6a-GFP-kanMX6	[3]
**PA239**	pFA6a-13myc-kanMX6	[3]
**PA241**	pFA6a-3HA-kanMX6	[3]
**PA242**	pFA6a-GFP-HIS3MX6	[3]
**PA410**	pRS303-peIF5A-GFP-TIF51A-HIS3MX6	Dr. Brian M. Zid
**PA431**	pRS303-peIF5A-GFP-TIF51A^{K51R}-HIS3MX6	This study
**PA432**	pRS303-peIF5A-GFP-eIF5AΔNLS-HIS3MX6	This study

Supplementary Table S3. Oligonucleotides used in this study

Primer	Sequence (5'-3')	
**Gene expression detection by RT-qPCR and ChIP-qPCR**		
ACT1-F	TCGTTCCAATTTACGCTGGTT	RT-qPCR/ChIP-qPCR
ACT1-R	CGGCCAAATCGATTCTCAA	RT-qPCR/ChIP-qPCR
BNI1-F	ACATGTGGAAAACGGAAAGC	RT-qPCR/ChIP-qPCR
BNI1-R	AGATCTTCTGCGCCATCTGT	RT-qPCR/ChIP-qPCR
BNR1-F	CCAGCTCCACCTTTACCAAA	RT-qPCR/ChIP-qPCR
BNR1-R	CCCAGTGGATTTGCTTCAAT	RT-qPCR/ChIP-qPCR
CLN2-F	TCAAAGCCACACTCCAATGA	RT-qPCR
CLN2-R	TTGCTGTTAGGACCCGTGA	RT-qPCR
GAL1-F	TGGTGTTAACAATGGCGGTA	ChIP-qPCR
GAL1-R	GGGCGGTTTCAAACTTGTTA	ChIP-qPCR
GAPDH-F	ATGACCGCCACTCAAAAGAC	RT-qPCR/ChIP-qPCR
GAPDH-R	CTTAGCAGCACCGGTAGAGG	RT-qPCR/ChIP-qPCR
Intergenic-F	GGCTGTCAGAATATGGGGCCGTAGTA	ChIP-qPCR
Intergenic-R	CACCCCGAAGCTGCTTTCACAATAC	ChIP-qPCR
LDB17-F	AACTCAAGCCTGGTTGCCTA	RT-qPCR/ChIP-qPCR
LDB17-R	CATCCGGTAGAGGTCACGAT	RT-qPCR/ChIP-qPCR
tetO-TIF51 qPCR	AATTACCGGATCAATTCGGG	RT-qPCR
TIF51A-1-R	TCGACAATCTTACATGGTCT	RT-qPCR
TIM50-F	TCTGCGTTGACAGGTACTGC	RT-qPCR/ChIP-qPCR
TIM50-R	AATCAGGGAAAGGTGGCTCT	RT-qPCR/ChIP-qPCR
TUP1-F	AAACCACTCTAAACCTATCC	RT-qPCR
TUP1-R	AGTATTCACCATCGTTACTG	RT-qPCR
RDN5-F	GTTGCGGCCATATCTACCAG	RT-qPCR
RDN5-R	AGCACCTGAGTTTCGCGTAT	RT-qPCR
VRP1-F	GGCAGAAATTAATGCCAGGA	RT-qPCR/ChIP-qPCR
VRP1-R	GTGGTGCAGTAGGCGGTAAT	RT-qPCR/ChIP-qPCR
YTA12-F	GTTTGTTGGTGTTGGTGCAG	RT-qPCR/ChIP-qPCR
YTA12-R	TCATCATTGGCACCTGAAAA	RT-qPCR/ChIP-qPCR
**Gene tagging by PCR**		
BNR1-F2	TTACTAGAGAGAACGCATGCTATGCTGAACGATATTCAAAATATACGGATCCCCGGGTTAATTAA	
BNR1-R1	TTTCTTTATATAAGCTCCACAACTACATAAAATACTAAGTCTTCAGAATTCGAGCTCGTTTAAAC	
CTK1-Tagging-F	AATAGTAATAATAATAATAATAATAATAATGACGATGATGATAAACGGATCCCCGGGTTAATTAA	
CTK1-Tagging-R	TTAATCTATTTTTTGTGTCTACTTATTTCAATTGGCTATATATCCGAATTCGAGCTCGTTTAAAC	
GAPDH-F2	TACTCCGCCAGAGTTGTTGACTTGATCGAATATGTTGCCAAGGCTCGGATCCCCGGGTTAATTAA	
GAPDH-R1	TGTATATTCAAAAAAAAATCATTATCCTCATCAAGATTGCTTTATGAATTCGAGCTCGTTTAAAC	
GFP-eIF5A-F	TAGACTCCCAAACACACACAAATACCAACTCATATATACACTAGTACACTCTATATTTTTTTATG	
GFP-eIF5A-R	TCTTTTTTCATTTATATCCCATGCCATGATGTTAACCGGTTTAATCGGTTCTAGCAGCTTCCTTG	
LDB17-F2	CCGCCTCCTCCTCCTCCTCCATCAAGAAAATGTGGAACTCCAAAACGGATCCCCGGGTTAATTAA	

LDB17-R1	ATGGTCGGAAGAACCACATTAATGCAAGAAGAAATAATGCTTTACGAATTCGAGCTCGTTTAAAC
TIM50-F2	TTATTTGAAGAGGAAAAGAAAAAAGAAGAAGATTGCTGAATCCAAACGGATCCCCGGGTTAATTAA
TIM50-R1	CACACATAGATACGTAGATACATGAGAAGAGGGTTTACATGAAAAGAATTCGAGCTCGTTTAAAC
YTA12-F2	GAAGAAAAAAACGAAAAACGTAATGAGCCTAAGCCATCTACAAACCGGATCCCCGGGTTAATTAA
YTA12-R1	ATATGTAGAACAGTCTTCCTCCATTTCTTTGTATTGTGAAATATCGAATTCGAGCTCGTTTAAAC
**Proline deletion/insertion by PCR**	
BNR1-delPro-F	AGTCTTGATAATGGAATCCAACTAGTACCTGAAGTTGTTAAACTATCCTTGTCGATGAACAAA
BNR1-delPro-R	TTTCTTTATATAAGCTCCACAACTACATAAAATACTAAGTCTTCAGAATTCGAGCTCGTTTAAAC
TIM50-delPro-F	CCTACTTCCAAGAGCCACCTTTCCCTGATTTACTACCAAAGGCCATTAACTCTTG
TIM50-delPro-R	CACACATAGATACGTAGATACATGAGAAGAGGGTTTACATGAAAA
YTA12-delPro-F	GCTACTTTGAAGGTAACAATAGCAGAAATATTCCACTAAATGATCCTAGTAATCC
YTA12-delPro-R	TTTCTTTATATAAGCTCCACAACTACATAAAATACTAAGTCTTCA
GAPDH-10ProNt-F3	CCACCGCCTCCTCCTCCTCCTCCTCCACCCTTCGGTAGAATCGGTAGATT
GAPDH-10ProNt-F4	CACTAAATTTACACACAAAACAAAATGATCAGAATTGCTATTAACGGTCCACCGCCTCCTCCTCCTCC
GAPDH-15Pro-R1	TGTATATTCAAAAAAAAATCATTATCCTCATCAAGATTGCTTTATGAATTCGAGCTCGTTTAAAC
**Targeted mutagenesis of PA410 plasmid**	
K51R-F	TAAGACTGGTCGCCACGGTCACG
K51R-R	GAAGTGGACATGTCG
ΔNLS-F	GGATGAACTATACAACCAATGCAATGTTCTGCCTTGAGAA
ΔNLS-R	TTGTATAGTTCATCCATGCCATGTG

Supplementary Table S4. Antibodies used in this study

Primary Antibody	Working dilution	Source	Secondary Antibody	Working dilution	Source
**Protein detection by Western blotting**					
eIF5A	1:500	Abcam (Ab32407)	α-rabbit	1:10000	Promega
G6PDH	1:15000	Roche (10127671001)	α-rabbit	1:10000	Promega
HA	1:5000	Roche (12013819001)	-	-	-
hyp-eIF5A	1:600	Genentech (FabHpu)	α-rabbit	1:10000	Promega
Myc	1:1000	Invitrogen (13-2500)	α-mouse	1:10000	Promega
GFP	1:5000	Merck (11814460001)	α-mouse	1:10000	Promega
H4	1:1000	Abcam (Ab7311)	α-rabbit	1:10000	Promega
PGK1	1:10000	ThermoFisher (22C5D8)	α-mouse	1:10000	Promega
**Chromatin immunoprecipitation**					
eIF5A	1:4	Abcam (Ab32407)	Dynabeads anti-rabbit IgG		Invitrogen (11203D)
Rpb1 (8WG16)	1:7	Invitrogen (MA1-10882)	Dynabeads Pan Mouse IgG		Invitrogen (11041)

2. Supplementary Materials and methods

Yeast strains, plasmids, and growth conditions

All *Saccharomyces cerevisiae* strains and plasmids used herein are listed in Supplementary Tables S1 and S2 respectively. For all the experiments carried out *S. cerevisiae* cells were grown in liquid YPD (2% glucose, 2% peptone, 1% yeast extract).

Plasmid pRS303-peIF5A-GFP-TIF51A-HIS3MX6 (PA410) and derivatives were used for expression of a second copy of eIF5A fused to GFP and subsequent visualization under microscopy. Plasmids pRS303-peIF5A-GFP-TIF51A^{K51R}-HIS3 (PA431) and pRS303-peIF5A-GFP-TIF51AΔNLS-HIS3MX6 (PA432) were constructed from the plasmid PA410, which was used as a template for PCR reaction using primers listed in Table S3 ("targeted mutagenesis" subsection). Parental plasmid was digested by restriction enzyme DpnI (Thermo Fisher Scientific) and the rest was transformed into bacteria for plasmid amplification and sequencing. The resulting plasmids were integrated into the genome of the corresponding strains by homologous recombination following the lithium acetate-based method [4] and transformants were selected in SC medium lacking histidine.

A PCR-based genomic tagging technique was employed to substitute the genomic full length eIF5A (*TIF51A*) ORF by eIF5A or eIF5AΔNLS fused to GFP at the N-terminal region. The plasmids PA410 and PA432 were used as templates for PCR reaction using primers listed in Table S3 ("gene tagging by PCR" subsection). The resulting cassettes were transformed in the corresponding strains following the lithium acetate-based method [4] and transformants were selected in SC medium lacking histidine. All the integrations were confirmed by genomic DNA conventional PCR.

A PCR-based genomic tagging technique was employed to tag the genomic full length *YTA12, TIM50, BNR1, LDB17* and *GAPDH* (*TDH1*) ORFs with GFP, HA or myc at the C-terminal region. The plasmids pFA6a-GFP-HIS3MX, pFA6a-GFP-KanMX, pFA6a-3HA-HIS3MX, pFA6a-3HA-KanMX, pFA6a-13myc-HIS3MX and pFA6a-13myc-KanMX (Longtine et al., 1998) were used as a template for PCR reaction using primers listed in Table S3 ("gene tagging by PCR" subsection). The resulting cassettes were transformed in the corresponding strains following the lithium acetate-based method [4] and transformants were selected in SC medium lacking histidine or YPD supplemented with geneticin. All the integrations were confirmed by genomic DNA conventional PCR.

To generate the strains harboring the deletion of the consecutive prolines from the *YTA12*, *TIM50* and *BNR1* gene sequences, the C-terminal *YTA12-GFP*, *TIM50-GFP* and *BNR1-3HA* sequences were amplified from genomic DNA of the strain PAY892, PAY888 and PAY896, respectively using primers listed in Table S3 ("proline deletion/insertion by PCR" subsection). The use of these primers resulted in the deletion of nucleotides 463-489 in *YTA12*, 541-561 in *TIM50* and 2305-2478 in *BNR1*, which encode for the 9, 7 and 25 prolines stretch of the Yta12, Tim50 and Bnr1 proteins respectively. The resulting PCR products were transformed in wild-type and *tif51A-1* strains as previously described and transformants were selected in SC medium lacking histidine. To generate the strains harboring the insertion of 10 consecutive prolines at the N-terminal of the *GAPDH* (*TDH1*) gene sequence, genomic DNA from PAY1443 yeast strain was used as template for PCR reaction using primers GAPDH-10ProNt-F3 and GAPDH-15Pro-R1 (Table S3 "proline deletion/insertion by PCR" subsection). The resulting PCR product was used as a template for a second PCR reaction using primers GAPDH-10ProNt-F4 and GAPDH-15Pro-R1 (Table S3 "proline deletion/insertion by PCR" subsection). The second PCR product was then transformed in wild-type and *tif51A-1* strains as previously described and transformants were selected in SC medium lacking histidine. All the insertions and deletions were confirmed by genomic DNA conventional PCR.

Experimental assays were performed with cells exponentially grown for at least four generations until required OD_{600} at the corresponding temperature. Temperature-sensitive strains were grown at the permissive temperature of 25°C until required OD_{600} and transferred to the non-permissive temperature of 37°C for 4 h for complete depletion of eIF5A but maintaining cell viability [5].

RT-qPCR analysis

For the analysis of the mRNA levels, total RNAs were isolated from yeast cells following the phenol:chloroform protocol. Briefly, a volume of an exponential phase culture corresponding to 10 OD_{600} units was harvested and flash frozen. Cells were resuspended in 500 µL of cold LETS buffer (LiCl 0.1 M, EDTA pH 8.0 10 mM, Tris-HCl pH 7.4 10 mM, SDS 0.2%) and transferred into a screw-cap tube already containing 500 µL of sterile glass beads and 500 µL of phenol:chloroform (5:1). Then, cells were broken using the Precellys 24 tissue homogenizer (Bertin Technologies) and centrifuged. The supernatant was transferred into a new tube containing 500 µL of phenol:chloroform (5:1) and then to a tube containing 500 µL of chloroform:isoamyl alcohol (25:1). RNA from the top phase was precipitated and finally dissolved in water for later quantification and quality control with Nanodrop device (Thermo Fisher Scientific).

The reverse transcription and quantitative PCR reactions were performed as detailed in Garre et al., 2013. Briefly, 2.5 µg of the total DNAse-I (Roche) treated RNA were retrotranscribed using an oligo d(T)18 with Maxima Reverse Transcriptase (Thermo Fisher Scientific). cDNA was labelled with SYBR Pre-mix Ex Taq (Tli RNase H Plus, Takara) and the Cq values were obtained from the CFX96 TouchTM Real-Time PCR Detection System (BioRad). Endogenous *ACT1* mRNA levels were used for normalization. At least three biological replicates of each sample were analyzed, and the specific primers designed to amplify gene fragments of interest are listed in Table S3.

Western blotting

For yeast protein content analysis by western blotting we followed the protocol described in [6]. Briefly, a cell culture volume corresponding to 10 OD_{600} units was harvested by centrifugation. For protein extraction, cell pellets were washed and resuspended in 200 µL of NaOH 0.2M and incubated at room temperature for 5 min for subsequent centrifugation at 12000 rpm for 1 min. Samples were then resuspended in 100 µL of 2X-SDS protein loading buffer (24 mM Tris-HCl pH 6.8, 10% glycerol, 0.8% SDS, 5.76 mM β-mercaptoethanol, 0.04% bromophenol blue) and boiled at 95°C for 5 min. After, lysates were centrifuged at 3000 rpm for 10 min at 4°C to remove cell debris and insoluble proteins, and supernatants were transferred into new tubes and stored at -20°C. Total protein content in the extract was quantified by an OD_{280} estimation in a Nanodrop device (Thermo Fisher Scientific) to load equal protein amounts per sample into the SDS-PAGE gel. The acrylamide percentage of the used SDS-PAGE depended on the molecular weight of the protein of interest.

SDS-PAGE and Western blotting were performed using standard procedures (BioRad). Blotting membranes were blocked with 5% skimmed milk in TBS-T (150 mM NaCl, 20 mM Tris, 0.1% Tween20, pH 7.6) for 1 h at room temperature and incubated with primary antibodies overnight at 4°C against either Myc, GFP, eIF5A, hyp-eIF5A, HA, H4, PGK1 or glyceraldehyde-6-phosphate dehydrogenase. Detailed information of the antibodies used in this study can be found in Table S4. Bound antibodies were detected using the appropriate horseradish peroxidase-conjugated secondary antibodies. Chemiluminiscent signals were detected with an ECL Prime Western blotting detection kit (GE Healthcare) and digitally analyzed using ImageQuant LAS 4000 software (GE Healthcare). In order to capture variation across all samples, the signal in each lane was normalized to the mean signal across all lanes in a single blot. Then, the resulting signal of bands was normalized against the corresponding G6PDH resulting signal. At least three biological replicates of each sample were analyzed.

Fluorescence microscopy and analysis

Yeast cells were grown to a logarithmic phase in YPD medium, centrifuged, washed and subjected to standard fluorescence and phase contrast microscopy. Fluorescence images were acquired using an Axio Imager Z1 fluorescence motorized microscope equipped with a Plan

Apochratic x63/1.4 oil-immersion objective and a 100 W mercury lamp (Carl Zeiss, Germany). Images were recorded with an AxioCam MRm digital camera (Carl Zeiss, Germany). To study nuclei localization, cells were incubated with 1 µg/mL 4′,6-Diamidino-2-phenylindole dihydrochloride (DAPI, Thermo Fisher Scientific) for 5 mins in the dark, washed and subjected to microscope. The following excitation and emission wavelengths were used: DAPI (excitation 359 nm; emission 457 nm) and GFP (excitation 475 nm; emission 509 nm). The same exposure times were used to acquire all images and at least three biological replicates of each sample were analyzed.

All the imaging analysis was performed on Image J software. For the analysis of fluorescence intensity signal ROIs were manually outlined around cells using the "Freehand" selection tool in ImageJ on DIC images. After background subtraction, the amount of GFP-eIF5A, its different versions and eIF4e-GFP (total fluorescence intensity) was quantified in the whole cell and in the nuclei section using the corresponding DAPI staining images. At least 100 single cells were scored from three independent experiments.

Chromatin immunoprecipitation

For eIF5A and Rpb1 chromatin binding experiments, yeast cell cultures were grown at 25°C in YPD and transferred to 37°C for 4 h when using temperature-sensitive strains. For the cross-linking reaction, formaldehyde was added (1% final concentration) to a volume of 45 mL of culture for 15 min at room temperature with occasional inversion. Then, the reaction was stopped with glycine (0.14 M) incubation for 5 min at room temperature. The chromatin immunoprecipitation (ChIP) experiments were performed as previously described [7] with the following modifications: after reversing formaldehyde-mediated cross-linking, samples were treated with proteinase K, and DNA was purified using the GeneJET PCR Purification Kit (Thermo Fisher Scientific, #K0702) according to the manufacturer's instructions. To determine the enrichment of the DNA regions bound by the protein of interest, qPCR was run as described above using the primers listed in Table S3. The qPCR amplification data were normalized with the total input DNA value in the corresponding whole cell extract. Detailed information of the antibodies and dynabeads used can be found in Table S4.

At least three biological replicates of each sample were analyzed.

ChIP-seq and sequencing analysis

Wild-type yeast cells exponentially grown in YPD at 25°C were used for ChIP-seq experiments. The concentration of the DNA samples (inputs and IPs) was quantified with Qubit dsDNA HS kit (Invitrogen) and fragment size distribution of the inputs was assessed on a TapeStation using the D5000 HS assay (Agilent). Libraries for ChIP-Seq were prepared at IRB Barcelona Functional Genomics Core Facility. Briefly, dual-indexed DNA libraries were generated from 2.1 – 3.1 ng of DNA samples using the NEBNext Ultra II DNA Library Prep kit for Illumina (New England Biolabs). 16 cycles of PCR amplification were applied to all libraries.

The final libraries were quantified using the Qubit dsDNA HS assay and quality controlled with the Bioanalyzer 2100 DNA HS assay (Agilent). An equimolar pool was prepared with the four libraries and submitted for single end 50 nt sequencing on a NextSeq2000 (Illumina). More than 4 Gbp of reads were produced, with a minimum of 17 million of single end reads per sample.

Data analyses was performed at the Statistical and Omics Data Analyses facility of the SCSIE-Universitat de València. Raw reads were quality-checked and trimmed to remove adapters and low-quality bases (Phred score ≥ 28 and reads ≥ 40 bp) using fastp (version v0.23.1) [8] and FastQC (version v0.11.5) tools. Processed reads were aligned using Hisat2 (version v2.2.1) [9] and *S. cerevisiae* genome (R64-1-1) (Ensembl release 110). Alignments were processed by SAMtools (version v1.13) [10] and alignment quality will be evaluated with QualiMap (version v2.2.2d) [11]. HTSeq v2.0.4 was used to generate raw counts per gene, using the 'gene_id'

attribute from exon features in the GTF annotation file (Saccharomyces_cerevisiae.R64-1-1.110.gtf) [12]. Enrichment graphic were obtained with ngs.plot (version v2.61) [13].

The eF5A binding value for each individual gene was calculated as the relative value respect the mean value for all genes.

Biological process Gene Ontology (GO) terms overrepresented in genes with the highest eIF5A binding or the highest eIF5A translation dependence measured with the Protein Pause Index (PPI). Gene set enrichment analysis was done using the ReviGO software.

Protein Pause Index (PPI) calculation

The dependency of each yeast gene on eIF5A for translation of its mRNA was estimated by calculating the PPI. First, the number of the top 43 eIF5A-dependent tripeptide motifs present in the encoded protein amino acid sequence was determined. These motifs cause ribosome pausing when eIF5A is depleted in yeast cells, as described by [14]. Secondly, the number of each motif was multiplied by its pause strength value, as revealed by 5PSeq analysis [14]. Thirdly, the PPI for each gene was obtained as the sum of motifs x strength.

Determination of individual transcription rates and mRNA levels

To determine the synthesis rate (TR) in the corresponding strains, a genomic run-on (GRO) was performed as originally described in [15]. Briefly, wild-type and *tif51A-1* cells were grown to early mid-log phase at 25°C and then transferred to 37°C for 4 h. Culture volumes were adjusted so that approximately $6x10^8$ cells were harvested to perform the run-on. A second sample corresponding to 20 mL of cell culture was also harvested for total RNA extraction. Both pellets were flash frozen and stored at -20°C. Upon defrosting, the cell pellet was washed in cold water and resuspended in 1 mL of 0.5% cold N-lauryl sarcosine sodium sulfate (sarkosyl) and transferred to a new tube. After permeabilization, cells were centrifuged at 6000 rpm for 1 min and the supernatant was removed. To perform the run-on, cells were resuspended with 115 µL of distilled water, 120 µL of 2.5x transcription buffer, 16 µL of ACG mix, 6 µL of 0.1 M DTT and 20 µL of [α-33P]-UTP (3000 Ci/mmol). The mixture was incubated at 30°C for 5 min and 600 rpm shaking to allow transcription elongation. The transcription was stopped with cold distilled water and the cells were collected by centrifugation at 6000 rpm for 1 min to remove the non-incorporated radioactive nucleotide. Then, total RNA was isolated following the phenol:chloroform protocol. A 5 µL aliquot was used for specific radioactivity determination using the Tricarb scintillation counter (Perkin Elmer). Home-made macroarray nylon filters [15] were pre-hybridized for 1 h with hybridization solution at 65°C. 300 µl of 2x hybridization solution were added to all the *in vivo* labelled RNA samples and mixed with 3 mL of hybridization solution. Macroarray filters were hybridized for 48 h in a roller oven at 65°C. The macroarrays were vacuum-sealed with plastic and exposed to an imaging plate (BAS-MP, FujiFilm) for the desired time depending on the signal intensity (1-7 days). The imaging plate was read at 50 µm resolution in a phosphorimager scanner (FLA-3000, FujiFilm).

For the second cell aliquot, total RNA was isolated following the phenol:chloroform protocol. Approximately 50 µg of RNA were purified using the Quiaquick kit (Qiagen) following manufacturer's instructions and used for reverse transcription into cDNA. For that, 200 U of Maxima Reverse Transcriptase (200 U/µL), 3 µL of oligo d(T)15VN (500 ng/µL), 1 µL of RNaseOUT, 3 µL of 0.1 M DTT, 6 µL of 5x RT buffer, 1.5 µL of dNTP mix, and 4 µL of [α-33P]-dCTP (3000 Ci/mmol) were added to a final volume of 30 µL. The labelling reaction was incubated for 2 h at 50°C and the reaction was stopped by adding 1 µL of 0.5 M EDTA. The labelled cDNAs were purified by a S300-HR MicroSpin column (Amersham Biosciences) so the non-incorporated radioactive nucleotide was removed. The hybridizations were performed as described previously for GRO except that cDNA samples were denatured at 95°C for 5 min prior hybridization and a final concentration of 3.5 x 106/mL was employed. Macroarray filters were hybridized for 24 h in a roller oven at 65°C. Both GRO and cDNA labelled samples belonging to the same sampling were successively hybridized against the same filter.

For the quantification of hybridization signals and subsequent analysis procedures: the experiments were always done in triplicate to reduce the variability provided by differences in *in vivo* incorporation, RNA extraction and hybridization. The scanned microarray images from both GRO and cDNA experiments were quantified using Array Vision software (Imaging Research) taking the sARM density (after background subtraction) as signal. Analysis of the data was performed as described [15]. For GRO and cDNA analysis, values that were at least 1.2 times higher than the local background were taken as valid measurements. An average data set of three replicates for each sample was created using median absolute deviation normalization by ArrayStat software (Imaging Research Inc.). Both GRO and cDNA hybridizations were normalized within each experiment replicate by the global mean procedure. Average cDNA values for each gene were finally corrected by percentage of guanines present in each probe-coding strand while average TR values for each gene were corrected by percentage of uridines present in each probe-coding strand.

The median values of the cell volumes of the population were obtained by a Coulter-Counter Z series device (Beckman Coulter, USA). We obtained the growth rates by growing 50 ml of yeast cultures in 250-ml flasks with shaking (190 rpm) at the corresponding temperature. Aliquots were taken every hour in the exponential phase and their OD_{600} (from 0.05 to 1.5) were measured. The generation time in the exponential phase were estimated from growth curves.

Chromatin association assay

The chromatin association assay experiment was performed as previously described in [16], with modifications. Yeast cell cultures were grown in 300 mL of YPD medium at 25°C to mid-log phase (OD_{600} 0.5). Subsequent steps were performed at 4°C with precooled buffers and in the presence of a fresh protease-inhibitor mix. Cells were collected by centrifugation, washed with 1×TBS buffer and with lysis buffer (150 mM NaCl, 50 mM HEPES-KOH pH7.5, 1 mM benzamidine, 1 mM PMSF, 1 mM EDTA, 1% TritonX-100, 0.1% sodiumdeoxycholate, 0.1% SDS and one protease inhibitor cocktail).

Cell pellets were flash frozen, thawed, resuspended in 1 mL lysis buffer, and disrupted via bead beating (FastPrep−24 Instrument, MP Biomedicals, LLC., France) in the presence of 0.5 mL of glass beads (MERCK, USA) for 40s at 4m/s, followed by an incubation of the sample for 1 min on ice. This was repeated eight times. The lysate was divided into two samples. One half was treated with 15 U of RNase A and 15 U of RNase T1 (Ambion, UK); the other half was treated with the same volume of the RNase storage buffer (10 mM HEPES pH 7.5, 1 mM EDTA, 0.1% Triton X-100, 50% glycerol). After 1 hour incubation at 25ºC, chromatin was isolated by centrifugation at 13,000 rpm for 20 min. This was repeated three times.

Chromatin was solubilized in 200 µL lysis buffer via sonication with a Bioruptor Standard Water bath Sonicator instrument (Diagenode). Chromatin solutions were then analysed by SDS-PAGE and Western blotting against eIF5A, cytoplasmic PGK1 and nuclear H4 proteins with specific antibodies. Detailed information of the antibodies used can be found in Table S4. At least three biological replicates of each sample were analyzed.

Supplemental References

1. Li Z, Vizeacoumar FJ, Bahr S, et al (2011) Systematic exploration of essential yeast gene function with temperature-sensitive mutants. Nat Biotechnol 29:361–367. https://doi.org/10.1038/nbt.1832

2. Barba-Aliaga M, Bernal V, Rong C, et al (2024) eIF5A controls mitoprotein import by relieving ribosome stalling at TIM50 translocase mRNA. J Cell Biol 223:e202404094. https://doi.org/10.1083/jcb.202404094

3. Longtine MS, McKenzie A, Demarini DJ, et al (1998) Additional modules for versatile and economical PCR-based gene deletion and modification in Saccharomyces cerevisiae. Yeast 14:953–961. https://doi.org/10.1002/(SICI)1097-0061(199807)14:10<953::AID-

YEA293>3.0.CO;2-U

4. Gietz D, Jean AS, Woods RA, Schiestl RH (1992) Improved method for high efficiency transformation of intact yeast cells. Nucleic Acids Res 20:1425. https://doi.org/10.1093/nar/20.6.1425

5. Li T, Belda-Palazón B, Ferrando A, Alepuz P (2014) Fertility and polarized cell growth depends on eIF5A for translation of polyproline-rich formins in Saccharomyces cerevisiae. Genetics 197:1191–1200. https://doi.org/10.1534/genetics.114.166926

6. Zuzuarregui A, Li T, Friedmann C, et al (2015) Msb2 is a Ste11 membrane concentrator required for full activation of the HOG pathway. Biochim Biophys Acta - Gene Regul Mech 1849:722–730. https://doi.org/10.1016/j.bbagrm.2015.02.001

7. Li T, De Clercq N, Medina DA, et al (2016) The mRNA cap-binding protein Cbc1 is required for high and timely expression of genes by promoting the accumulation of gene-specific activators at promoters. Biochim Biophys Acta - Gene Regul Mech 1859:405–419. https://doi.org/10.1016/j.bbagrm.2016.01.002

8. Chen S, Zhou Y, Chen Y, Gu J (2018) Fastp: An ultra-fast all-in-one FASTQ preprocessor. Bioinformatics 34:i884–i890. https://doi.org/10.1093/bioinformatics/bty560

9. Kim D, Paggi JM, Park C, et al (2019) Graph-based genome alignment and genotyping with HISAT2 and HISAT-genotype. Nat Biotechnol 37:907–915. https://doi.org/10.1038/s41587-019-0201-4

10. Danecek P, Bonfield JK, Liddle J, et al (2021) Twelve years of SAMtools and BCFtools. Gigascience 10:. https://doi.org/10.1093/gigascience/giab008

11. Okonechnikov K, Conesa A, García-Alcalde F (2016) Qualimap 2: Advanced multi-sample quality control for high-throughput sequencing data. Bioinformatics 32:292–294. https://doi.org/10.1093/bioinformatics/btv566

12. Putri GH, Anders S, Pyl PT, et al (2022) Analysing high-throughput sequencing data in Python with HTSeq 2.0. Bioinformatics 38:2943–2945. https://doi.org/10.1093/bioinformatics/btac166

13. Shen L, Shao N, Liu X, Nestler E (2014) Ngs.plot: Quick mining and visualization of next-generation sequencing data by integrating genomic databases. BMC Genomics 15:284. https://doi.org/10.1186/1471-2164-15-284

14. Pelechano V, Alepuz P (2017) EIF5A facilitates translation termination globally and promotes the elongation of many non polyproline-specific tripeptide sequences. Nucleic Acids Res 45:7326–7338. https://doi.org/10.1093/nar/gkx479

15. García-Martínez J, Aranda A, Pérez-Ortín JE (2004) Genomic run-on evaluates transcription rates for all yeast genes and identifies gene regulatory mechanisms. Mol Cell 15:303–313. https://doi.org/10.1016/j.molcel.2004.06.004

16. Battaglia S, Lidschreiber M, Baejen C, et al (2017) RNA-dependent chromatin association of transcription elongation factors and pol II CTD kinases. Elife 6:e25637. https://doi.org/10.7554/eLife.25637

S1A

Classification of genes into groups according to their eIF5A-dependency for translation

Protein Pause Index (PPI)	Gene number
0	1745
0-10	2454
10-20	1412
20-30	600
30-40	243
40-50	94
50-60	53
60-70	54
70-80	31
> 80	27

S1B

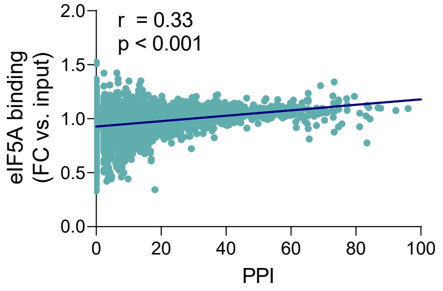

S1C

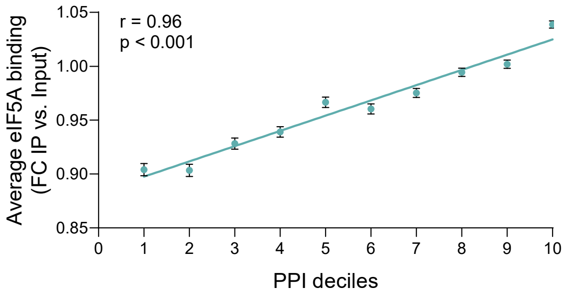

S1D

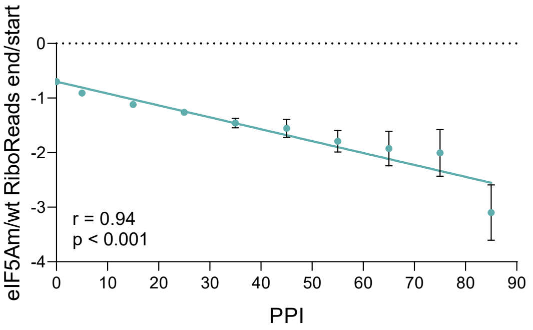

S1E

Genes selected for analysis

Gene	eIF5A-dependent motifs	PPP motifs	Protein Pause Index (PPI)
Intergenic	0	0	0
GAL1	2	0	9
ACT1	2	0	7
GAPDH	2	0	7
TIM50	9	5	48
LDB17	10	7	62
YTA12	12	7	73
BNR1	34	17	188
BNI1	39	21	217
VRP1	90	37	466

Supplementary Figure S1

Supplementary Figure S1. eIF5A chromatin binding positively correlates with the protein pause index (PPI)**. (A)** Classification of genes into groups with respect their putative dependency on eIF5A for their translation as determined by the different PPI intervals (see M&M for full definition of PPI). The number of genes included in each group is shown. **(B)** eIF5A binding values from ChIP-seq analyses were plotted against the protein pause index (PPI) value associated for each gene. Experimental data were adjusted to potential trend. The Pearson's correlation coefficient and the associated significance for the plot is shown. **(C)** The average eIF5A binding value relative to input from ChIP-seq analyses is shown for the genes included in each PPI decile. Genes were ranked according to their PPI values and divided into ten deciles containing an equal number of genes. Experimental data were adjusted to potential trend. The Standard error (SE) and Pearson's correlation coefficient and the associated significance for the plot is shown. **(D)** The average eIF5Am/wt RiboReads end/start calculated from data in (Schüller *et al*, 2017) is shown for the genes included in each PPI interval group (see M&M for full definition of eIF5Am/wt RiboReads end/start). Experimental data were adjusted to potential trend. The Standard error (SE), Pearson's correlation coefficient and the associated significance for the plot is shown. **(E)** Genes selected for ChIP analysis. Number of total eIF5A-dependent tripeptide motifs (Pelechano and Alepuz, 2017), polyproline motifs (PPP) and PPI indexes are shown.

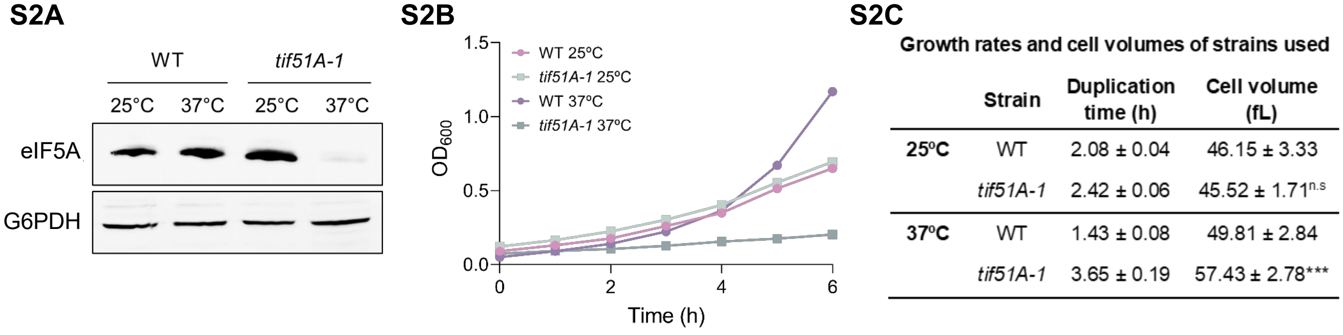

S2C

Growth rates and cell volumes of strains used

	Strain	Duplication time (h)	Cell volume (fL)
**25ºC**	WT	2.08 ± 0.04	46.15 ± 3.33
	tif51A-1	2.42 ± 0.06	45.52 ± 1.71$^{n.s}$
**37ºC**	WT	1.43 ± 0.08	49.81 ± 2.84
	tif51A-1	3.65 ± 0.19	57.43 ± 2.78***

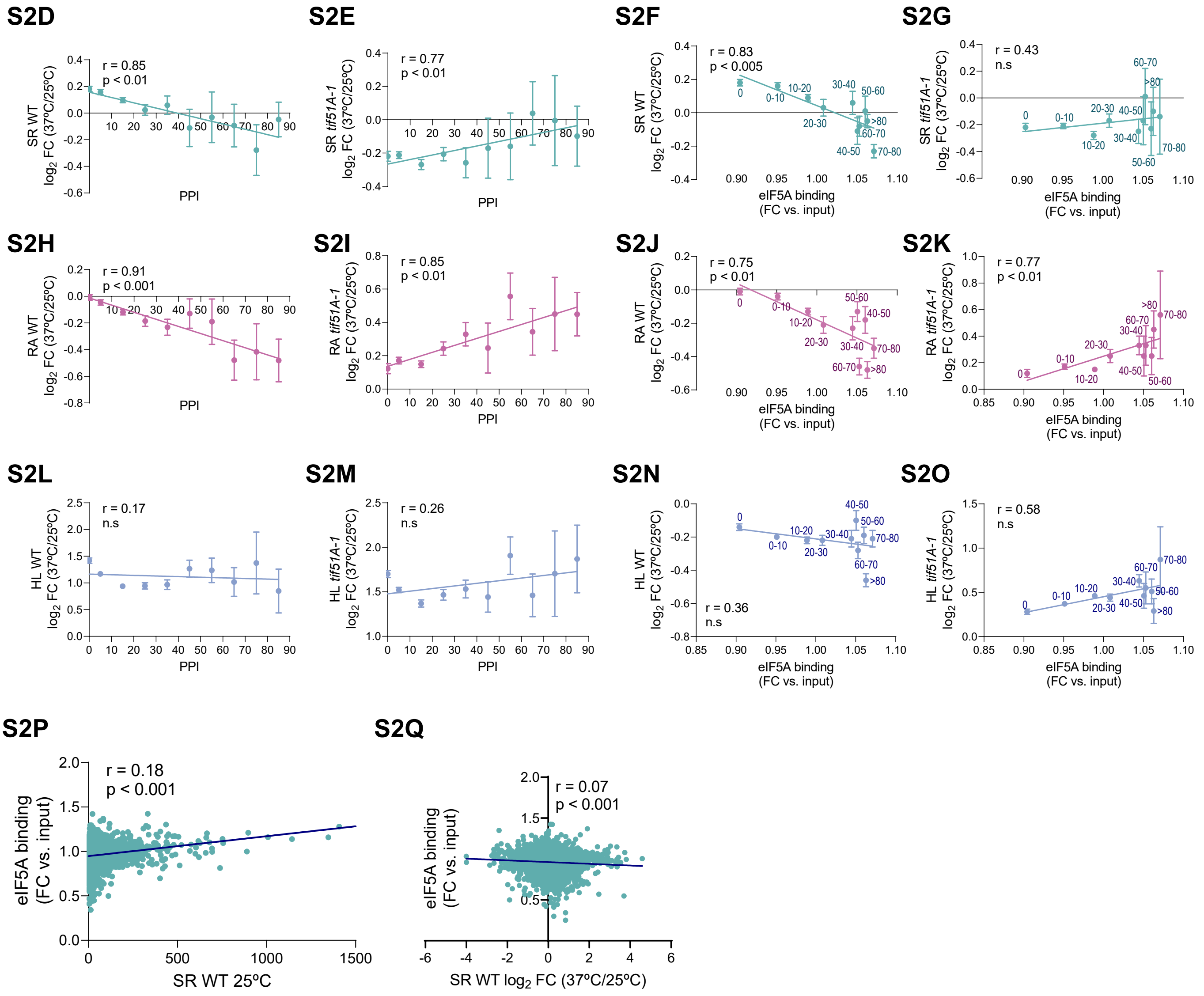

Supplementary Figure S2

Supplementary Figure S2. Changes in synthesis rate (SR) and RNA amount (RA) following eIF5A depletion are correlated with the extent to which translation is dependent on eIF5A and with eIF5A binding to chromatin. **(A,B,C)** eIF5A depletion causes changes in growth rate. Wild-type and *tif51A-1* mutant yeast strains were grown in YPD medium at 25ºC until early exponential phase and then transferred to 25ºC and 37ºC. **(A)** A representative Western blotting experiment from three independent experiments of the eIF5A protein levels in the wild-type and *tif51A-1* cells incubated at the indicated temperatures for 4 hours. G6PDH protein levels were used as loading control. **(B)** Growth measured as OD_{600} of the yeast cultures at the indicated time points. A representative experiment from three independent replicates is shown. **(C)** Duplication times were calculated from data in **(B)**. Cell volume was measured with a Coulter counter for each strain at the corresponding temperature. Statistical significance for volume differences was determined using a two-tailed paired Student's t-test relative to wild-type cells at 25ºC. ***p<0.001. n.s means no significant differences. **(D,E,H,I,L,M)** Graphs represent the average SR **(D,E)**, RA **(H,I)**, and half-lives (HL) **(L,M)** for all genes included in each Protein Pause Index (PPI) interval group. Values are given as the $\log_2$ Fold Change (FC) at 37ºC vs. 25ºC ± S.E. of wild-type or *tif51A-1* cells. **(F,G,J,K,N,O)** Graphs represent the average SR **(F,G)**, RA **(J,K)**, and HL **(N,O)** for all genes included in different PPI group (the labels indicate the PPI interval) versus the average eIF5A binding from ChIP-seq analyses for each group of genes. Values represent the $\log_2$ Fold Change (FC) at 37ºC vs. 25ºC ± S.E. of wild-type or *tif51A-1* strain. **(P)** eIF5A binding values from ChIP-seq analyses were plotted against the corresponding SR value from GRO analysis associated for each gene. **(Q)** eIF5A binding values from ChIP-seq analyses were plotted against the $\log_2$ Fold Change at 37ºC vs. 25ºC of SR of wild-type cells. **(D-Q)** Experimental data were adjusted to linear trends. Pearson's correlation coefficient and the associated significance for the plots are shown. n.s means no significant differences.

S3A

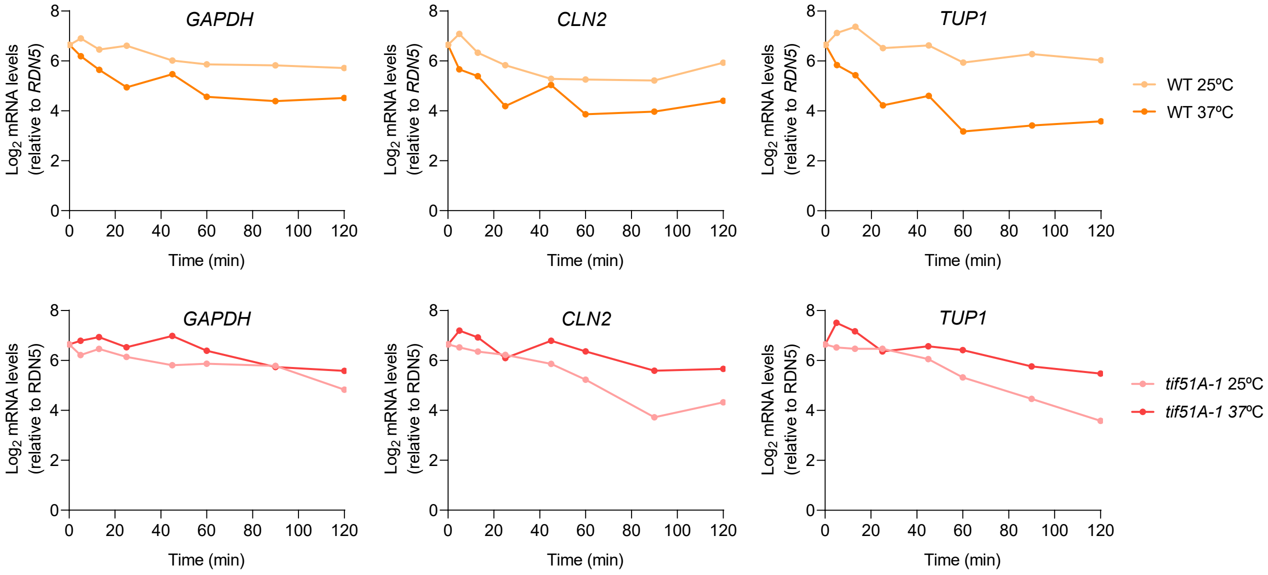

S3B

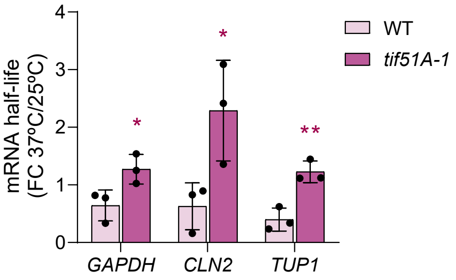

Supplementary Figure S3. mRNA stability increases upon eIF5A depletion. **(A)** mRNA decay kinetics of *GAPDH* (*TDH1*), *CLN2*, and *TUP1* in wild-type (WT) and *tif51A-1* mutant yeast strains. Cells were grown to mid-exponential phase in YPD at 25°C and then transferred to 25ºC or 37ºC for four hours. Transcription was inhibited by adding thiolutin (5 µg/mL) and samples were collected at 0, 5, 13, 25, 45, 60, 90 and 120 min following transcriptional shut-off. mRNA levels were determined by RT-qPCR, normalized to *RDN5* (5S rRNA) levels, and expressed as Log_2 mRNA levels relative to the 0 min time point. Graphs show the mean of three biological replicates. **(B)** mRNA half-life fold change (FC) 37°C vs. 25°C for *GAPDH*, *CLN2*, and *TUP1*. Half-lives were calculated from the decay curves shown in **(A)** by fitting the log2 of the data points to a linear regression model to calculate the slope which was then converted into half-life using the equation HL = 0.693/slope. Results are presented as individual values together with the mean ± SD from three independent experiments. Statistical significance was determined using a two-tailed unpaired Student’s t-test.

Supplementary Figure S3

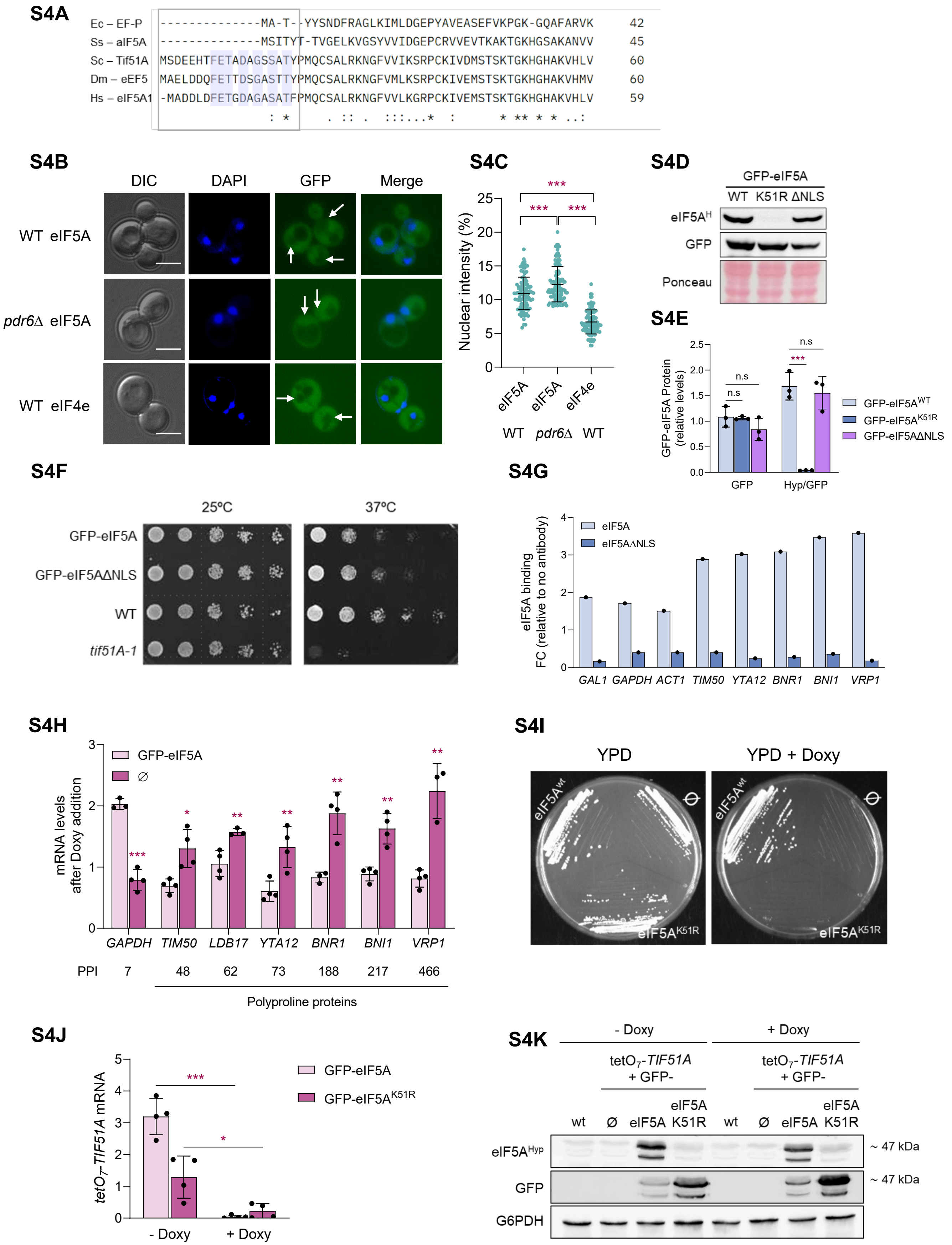

Supplementary Figure S4

Supplementary Figure S4. Localization, expression and functionality of yeast strains with modified eIF5A nuclear localization and cytoplasmic activity. **(A)** Amino acid sequence alignment of genes encoding EF-P in *E.coli* (Ec-EF-P), eIF5A in archaeal *Saccharolobus solfataricus* (S-aIF5A), Tif51A in *S. cerevisiae,* eIF5A in *Drosophila melanogaster* (Dm-eEF5) and eIF5A1 in *Homo sapiens* (H.s). Only the protein N-terminal regions are shown. Residues with an asterisk are conserved in all organisms and residues in purple are identical only in the eukaryotic eIF5A. The region containing the first 19 amino acid residues responsible for the nuclear localization is marked by a box. **(B)** Wild-type and *pdr6∆* strains expressing a second copy of GFP-eIF5A, and wild-type strain expressing the control eIF4e-GFP were cultured in YPD medium until reaching exponential phase and subjected to fluorescence microscopy. Cells were incubated for 5 mins with DAPI prior microscopy to stain the nuclei. White arrows indicate the nuclei. A representative image is shown from three independent experiments. Scale bar, 4 µm. **(C)** Quantification of percentage of nuclear signal is shown from a minimum of 100 cells. Results are presented as individual values together with the mean ± SD from three independent experiments. The statistical significance was measured by using a Kruskal-Wallis followed by Wilcoxon rank-sum analysis. **(D,E)** Wild-type strain with the expression of a second copy GFP-eIF5A^{WT}, GFP-eIF5A^{K51R} or GFP-eIF5A∆NLS was cultured in YPD at 25ºC. Proteins were extracted and analyzed by Western-blotting using anti-hypusinated-eIF5A and anti-GFP antibodies **(D)** and quantified **(E)**. **(E)** Data are shown as the mean relative protein level ± SD from three independent experiments. Statistical significance of protein was determined using a two-tailed paired Student's t-test. **(F)** Wild-type expressing GFP-eIF5A or GFP-eIF5A∆NLS in the *TIF51A* locus, wild-type and *tif51A-1* strains were cultured in YPD plates at the indicated temperatures. **(G)** ChIP analysis of eIF5A recruitment in wild-type expressing GFP-eIF5A or GFP-eIF5A∆NLS in the *TIF51A* locus and exponentially-grown in YPD at 25ºC. ChIP of eIF5A was performed using an anti-eIF5A antibody. The immunoprecipitated DNA was used to quantify the binding to different genes by qPCR using primers designed for amplification in the ORF regions. The percentage of the signal obtained in each ChIP sample with respect to the signal obtained with the DNA from the corresponding whole cell extract was calculated. One representative experiment is shown. **(H)** Yeast strains with tetO$_7$-*TIF51A* at the *TIF51A* locus and with a second copy of GFP-eIF5A or without second copy (Ø) were cultured overnight in YPD at 25ºC in the presence of doxycycline (15 µM) to deplete eIF5A expressed from the first copy, and then, mRNA levels from each gene were determined by RT-qPCR using primers designed for amplification in the ORF regions. Results are presented as individual values together with the mean ± SD from at least three independent experiments. Statistical significance was determined using a two-tailed unpaired Student's t-test. **(I)** Yeast strain with tetO$_7$-*TIF51A* at the endogenous locus and with a second copy of GFP-eIF5A, GFP-eIF5A^{K51R} or without second copy (Ø) were grown in YPD and then plated to test growth in YPD plates with or without doxycycline (15 µM). **(J,K)** Same yeast strains used in **(I)** were cultured overnight in YPD at 25ºC in the presence or absence of doxycycline (15 µM) and then, the tetO$_7$-*TIF51A* mRNA levels were determined by RT-qPCR using specific primers **(J)**, and the expression of the second copy GFP-eIF5A, of GFP-eIF5A^{K51R} was tested by Western blotting using anti-hypusinated-eIF5A, anti-GFP or anti-G6PDH antibodies. A wild-type strain was included as additional negative control **(K)**. **(J)** Results are presented as individual values together with the mean ± SD from at least three independent experiments. Statistical significance was determined using a two-tailed paired Student's t-test. For all experiments *p<0.05, **p<0.01, ***p<0.001. n.s means no significant differences.

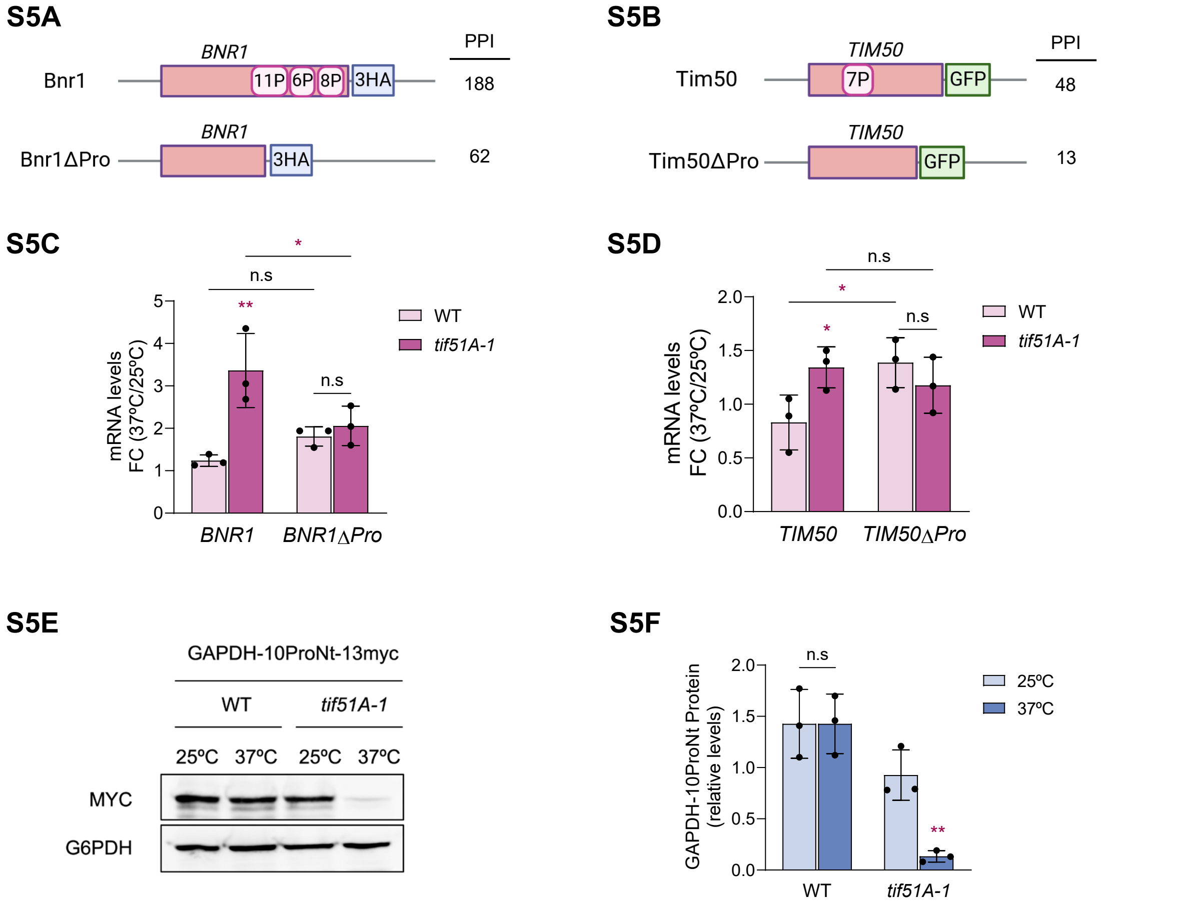

Supplementary Figure S5. Nucleotide sequences that encode polyPro motifs are necessary to promote transcriptional repression by eIF5A. **(A,B)** Schematic diagram showing the C-terminal genomic tagging of native *BNR1* **(A)** and *TIM50* **(B)** ORFs as well as their polyPro-deleted versions (*BNR1ΔPro, TIM50ΔPro*). PPI of both native and mutated versions are shown. **(C,D)** Exponentially growing cultures of wild-type and *tif51A-1* mutant yeast strains, carrying native and mutated versions of *BNR1* and *TIM50* genes were grown in YPD medium at 25ºC until exponential phase and then transferred to 25ºC and 37ºC for four hours. mRNA levels of the *BNR1* **(C)** and *TIM50* **(D)** versions were determined by RT-qPCR. **(C,D)** Data are presented as the mean fold change (FC) 37ºC vs. 25ºC ± SD from three independent experiments. Statistical significance was determined by using a two-way ANOVA followed by Estimated Marginal Means (emmeans) post-hoc tests. **(E,F)** Wild-type and *tif51A-1* yeast strains carrying a genomic *GAPDH* version with an N-terminal insertion of a polyPro sequence and a C-terminal tagging with myc (*GAPDH*-10ProNt-13myc, see schematic diagram in Fig.4D) were grown in YPD medium at 25ºC until exponential phase and then transferred to 25ºC and 37ºC for four hours. Protein levels of the Gapdh protein with the polyPro sequence were determined by Western blotting using anti-myc antibodies and anti-G6PDH as loading control **(E)**, and quantified **(F)**. **(F)** Data are shown as the mean relative protein level ± SD from three independent experiments. Statistical significance of protein level at 37ºC vs. 25ºC was determined using a two-tailed paired Student's t-test. For all experiments *p<0.05, **p<0.01, ***p<0.001. n.s means no significant differences.

Supplementary Figure S5

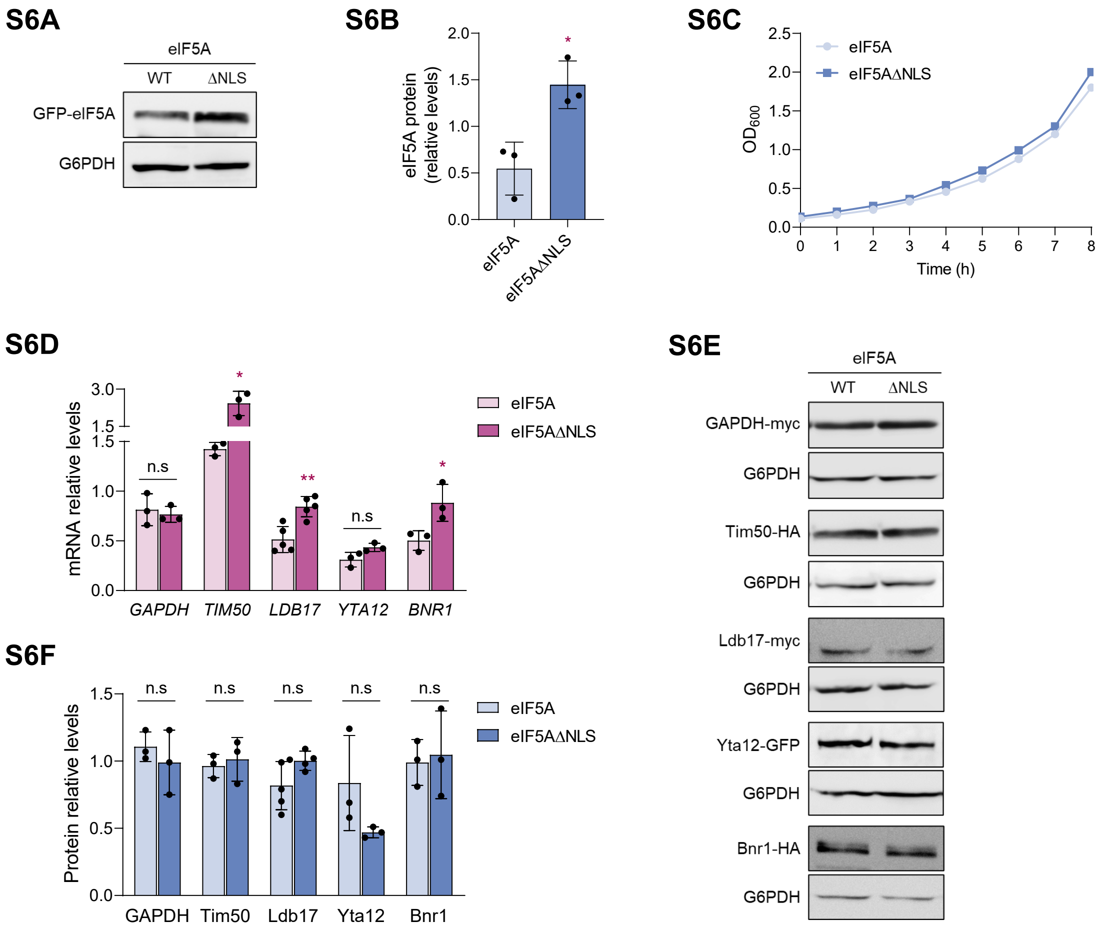

Supplementary Figure S6. Study of the mRNA and protein synthesis by an eIF5A mutant protein without the NLS region. **(A-F)** Wild-type cells expressing the native eIF5A or the eIF5AΔNLS version in the *TIF51A* locus were grown in YPD medium at 30ºC until exponential phase. eIF5A protein levels were determined by Western blotting **(A)** and quantified from three independent replicates **(B)** and growth was determined by measuring the OD_{600} of the yeast cultures at the indicated time points. A representative experiment is shown **(C)**. **(D)** mRNA levels from *GAPDH*, *TIM50*, *LDB17*, *YTA12* and *BNR1* genes were determined by RT-qPCR using primers designed for amplification in the specific ORF regions **(E,F)** The corresponding protein levels were determined by Western blotting **(E)** and quantified **(F)**. **(A,E)** G6PDH levels were used as loading control. A representative image is shown. **(B,D,F)** Data are presented as the mean ± SD from at least three independent experiments. Statistical significance was determined using a two-tailed unpaired Student's t-test relative to corresponding wild-type cells. $^*p<0.05$, $^{**}p<0.01$. n.s means no significant differences.

Supplementary Figure S6
